# Transcriptomic characterization of recombinant *Clostridium beijerinckii* NCIMB 8052 expressing methylglyoxal synthase and glyoxal reductase from *Clostridium pasteurianum* ATCC 6013

**DOI:** 10.1128/aem.01012-24

**Published:** 2024-09-11

**Authors:** Santosh Kumar, Eric Agyeman-Duah, Marian M. Awaga-Cromwell, Victor C. Ujor

**Affiliations:** 1 Department of Food Science, University of Wisconsin-Madison, Madison, Wisconsin, USA; Kyoto University, Kyoto, Japan

**Keywords:** methylglyoxal, iron uptake, transcriptomic analysis, NAD biosynthesis, signal transduction, butanol production, cysteine desaturase, *Clostridium beijerinckii*

## Abstract

Bioconversion of abundant lactose-replete whey permeate to value-added chemicals holds promise for valorization of this expanding food processing waste. Efficient conversion of whey permeate-borne lactose requires adroit microbial engineering to direct carbon to the desired chemical. An engineered strain of *Clostridium beijerinckii* NCIMB 8052 (*C. beijerinckii*_mgsA+mgR) that produces 87% more butanol on lactose than the control strain was assessed for global transcriptomic changes. The results revealed broadly contrasting gene expression patterns in *C. beijerinckii*_mgsA+mgR relative to the control strain. These were characterized by widespread decreases in the abundance of mRNAs of Fe-S proteins in *C. beijerinckii*_mgsA+mgR, coupled with increased differential expression of lactose uptake and catabolic genes, iron uptake genes, two-component signal transduction and motility genes, and genes involved in the biosynthesis of vitamins B_5_ and B_12_, aromatic amino acids (particularly tryptophan), arginine, and pyrimidines. Conversely, the mRNA patterns suggest that the L-aspartate-dependent *de novo* biosynthesis of NAD as well as biosynthesis of lysine and asparagine and metabolism of glycine and threonine were likely down-regulated. Furthermore, genes involved in cysteine and methionine biosynthesis and metabolism, including cysteine desulfurase—a central player in Fe-S cluster biosynthesis—equally showed reductions in mRNA abundance. Genes involved in biosynthesis of capsular polysaccharides and stress response also showed reduced mRNA abundance in *C. beijerinckii*_mgsA+mgR. The results suggest that remodeling of cellular and metabolic networks in *C. beijerinckii*_mgsA+mgR to counter anticipated effects of methylglyoxal production from heterologous expression of methylglyoxal synthase led to enhanced growth and butanol production in *C. beijerinckii*_mgsA+mgR.

**IMPORTANCE:**

Biological production of commodity chemicals from abundant waste streams such as whey permeate represents an opportunity for decarbonizing chemical production. Whey permeate remains a vastly underutilized feedstock for bioproduction purposes. Thus, enhanced understanding of the cellular and metabolic repertoires of lactose-mediated production of chemicals such as butanol promises to identify new targets that can be fine tuned in recombinant and native microbial strains to engender stronger coupling of whey permeate-borne lactose to value-added chemicals. Our results highlight new genetic targets for future engineering of *C. beijerinckii* for improved butanol production on lactose and ultimately in whey permeate.

## INTRODUCTION

With increasing global demand for cheese, the accumulation of whey—a byproduct of cheese production—is expected to continue to increase significantly (1). According to Kosikowski (1), ~9 kg of whey is generated per kilogram of cheese produced. Global cheese production stood at 25.3 million metric tons in 2023 (2), which corresponds to the accumulation of 227.7 metric tons of whey. The dairy industry deploys ultrafiltration to separate the protein content of whey, which is sold as a supplement (3–5). This leaves behind a lactose-rich effluent referred to as whey permeate (WP), which places a substantial economic burden on the industry, while posing a hazard to the environment (4–6). With biochemical oxygen demand and chemical oxygen demand ranging from 40,000 to 48,000 mg/L and 80,000 to 95,000 mg/L, respectively (4, 7), land spreading of WP constitutes a major hazard to surface water bodies (4–6). Although WP is used to compound livestock feed, this practice is dwindling, thus encumbering the dairy industry with WP treatment costs. Spray drying to produce whey powder incurs additional costs, which makes this approach economically less attractive (4, 8). Consequently, developing cost-effective strategies for the valorization of WP represents considerable economic and environmental payoffs.

Toward valorization of WP, we previously sought to engineer *Clostridium beijerinckii* NCIMB 8052 (hereafter *C. beijerinckii*) to co-produce 1,2-propanediol (1,2-PDO) and n-butanol (hereafter, butanol) on lactose (WP sugar). *C. beijerinckii*_mgsA+mgR was engineered by fusing methylglyoxal synthase (MgsA) and methylglyoxal reductase (MgR) from *Clostridium pasteurianum* using a polyglycine linker, under the control of an acetoacetate decarboxylase promoter. The fused construct was ligated into pWUR459 and then transformed into *C. beijerinckii*. MgsA and MgR were fused such that the protein product of MgsA (i.e., methylglyoxal), which is a toxic intermediate, would be readily metabolized (detoxified) by the adjoining MgR. The control strain (*C. beijerinckii*_p459) was obtained by transforming *C. beijerinckii* with the empty plasmid. Although *C. beijerinckii*_mgsA+mgR did not produce 1,2-PDO, it exhibited 35% more growth and produced at least 87% more butanol than *C. beijerinckii*_p459 (9). Both 1,2-PDO and butanol are value-added chemicals with multifarious applications across the economy and are currently produced solely from fossil fuel feedstocks. Butanol has enormous potential as renewable fuel for aviation and automobile applications, as a feedstock in the production of plasticizers, synthetic rubber, and paints and as a broad-application industrial solvent (10–12). Similarly, 1,2-PDO finds application in the manufacture of polyester resins, building materials, non-ionic detergents, and cosmetics and as a pharmaceutical solvent (13–16).

In this study, we sought to understand the molecular underpinnings of enhanced butanol production by *C. beijerinckii*_mgsA+mgR relative to the empty plasmid control strain (*C. beijerinckii*_p459). Using RNA sequencing, we profiled the global mRNA levels in *C. beijerinckii*_mgsA+mgR grown on 50 g/L lactose in comparison to *C. beijerinckii*_p459. Transcriptomic results suggest that *de novo* NAD biosynthesis via L-aspartate was strongly down-regulated in *C. beijerinckii*_mgsA+mgR. Consistent with this observation, cultures of *C. beijerinckii*_mgsA+mgR supplemented with aspartic acid (2 g/L), showed reduced growth and butanol production. We discuss the implications of observed mRNA profiles in this strain, in relation to the observed solvent profiles during lactose catabolism with and without aspartic acid supplementation.

## MATERIALS AND METHODS

### RNA library construction and sequence analysis

We have previously observed that cloning and co-expression of *mgsA* and *mgR* from *C. pasteurianum* in *C. beijerinckii* resulted in a significant increase in butanol production (9). In this study, using RNA sequencing, we sought to delineate the underlying molecular basis for increased butanol production in the recombinant strain (*C. beijerinckii*_mgsA+mgR) relative to the empty plasmid control (*C. beijerinckii*_p459). To isolate total RNA, *C. beijerinckii*_p459 and *C. beijerinckii*_mgsA+mgR were grown in P2 medium as previously described (12), with a slight modification. In place of 60 g/L glucose, the P2 medium contained 50 g/L lactose as carbon source. All cultures were supplemented with erythromycin (15 µg/mL). Because of the varying growth rates between both strains (*C. beijerinckii*_mgsA+mgR and *C. beijerinckii*_p459), cell samples for RNA isolation were taken when the cultures of each strain reached an optical density (OD)_600_ of ~1.0. Triplicate cell samples (10 mL) were collected for RNA isolation. The cells were centrifuged for 10 min at 4,000 × *g*. Afterwards, total RNA was extracted with TRIzol according to a previously reported method (12). DNA contamination was removed by DNase I treatment (NEB, USA). The quality and quantity of RNA samples were assessed using a NanoDrop One UV-Vis Spectrophotometer (Thermo Fisher Scientific, Waltham, MA, USA). We have previously established a protocol for normalizing RNA (12), which was used in this study. This entailed defining the conditions and duration for DNase treatment to achieve complete DNA removal from *C. beijerinckii* RNA samples. Furthermore, PCR amplification of RNA polymerase sigma factor (*rpoD*) was set up using the RNA samples as template following DNase treatment, to confirm complete removal of DNA contamination. Ribosomal RNA (rRNA) depletion, library preparation, mRNA sequencing, and initial data processing were performed commercially by SeqCoast Genomics (Portsmouth, NH, USA). In brief, rRNAs were removed with the Ribo-Zero Plus Microbiome, and then, unique dual index libraries were generated. Sequencing was performed on the Illumina NextSeq2000 platform using a 300-cycle flow cell kit to produce 50 M 2 × 150 bp paired reads. Read de-multiplexing, read trimming, and run analytics were performed using DRAGEN v3.10.12, an on-board analysis software on the NextSeq2000. Sequencing quality control was assessed by FastqQC metrics. The reads trimming tool Trimmomatic (version 0.39; 17) was used to remove adaptors and low-quality bases and reads, and Kallisto version 2.1.2 (18) was used to generate count tables and normalized expression levels for each of the genes from the reference genome (*C. beijerinckii* NCIMB 8052). DeSeq2 version 2.23.0 (19) was employed to estimate differentially expressed genes and compare gene expression levels in *C. beijerinckii*_mgsA+mgR versus *C. beijerinckii*_p459. Volcano plot was generated using EnhancedVolcano version 2.3, package R (20). BAKTA version 1.5.1 (21) was used for functional annotation of genes. The mRNAs with fold changes ≥ 1.2 (Log_2_; equivalent to fold changes ≥ 2.3 Log_10_) and a *P* value < 0.05 were considered to be differentially expressed. The mRNA fold changes are presented in Log_2_.

### Real-time quantitative PCR

For the real-time quantitative PCR (RT-qPCR) assay, RNA was isolated as described above from triplicate cultures of *C. beijerinckii*_mgsA+mgR and *C. beijerinckii*_p459 grown on lactose and supplemented with erythromycin (15 µg/mL). Using the RNA as template, cDNA was synthesized using the iScript cDNA Synthesis Kit (Bio-Rad Inc., Hercules, CA, USA). RT-qPCR was performed as per the method described previously (12) using gene-specific primers (Table 1) and iTaq Universal SYBR Green Supermix (Bio-Rad, Hercules, CA, USA) in a CFX Connect RT-qPCR System (Bio-Rad, Hercules, CA, USA). Relative expression was normalized relative to those obtained for the *rpoD* housekeeping gene (Cbei_0853) of *C. beijerinckii*, and the fold change of selected genes was calculated using the 2^−ΔΔCt^ method. The data presented are average values of three biological replicates ± standard deviation.

**TABLE 1:** List of primers used for RT-qPCR analysis

Primer	Sequences (5′−3′)	Gene function/name
Cbei_0792F Cbei_0792R	GCACACTATTACCAACCAGGA CTGCACTAGGACATGGCATTA	Quinolinate synthetase (*nadA*)
Cbei_0794F Cbei_0794R	TGCGGCTGGTGGAATAAA ATCTGCGCCTGCTAATAGTG	Nicotinate-nucleotide pyrophosphorylase/quinolinate phosphoribosyl transferase (*nadC*)
Cbei_0793F Cbei_0793R	AGGCGCTCATTCTGTAAATAGG TATGCCTCCAGTTGCCAATAC	L-Aspartate oxidase (*nadB*)
Cbei_0620F Cbei_0620R	GCCCATCCCTTAGCACTTATT GCCTAATACTCTGCCAATCTCTC	Oxidoreductase/nitrogenase (*nifD1*)
Cbei_2281F Cbei_2281R	CCTGAATTTATAGCAGGGCAATTTA CCATTCTCTTCTCTCTTTACACTTATTC	Oxidoreductase FAD/NAD(P)-binding protein
Cbei_1546F Cbei_1546R	TTGGAATAACATTCCTAACCAAAGG GCATTATCGCAGGCGAATTT	Hypothetical protein
Cbei_3015F Cbei_3015R	GCTCAGCTTATGGATCAGAAGAA GCACCATATGACTCCATTCTCTT	Flavodoxin
Cbei_3778F Cbei_3778R	GGCAGGAGCAGAAGTAGTAAAT ATGCTGACCATCACCATCTATC	Glycosyl transferase
Cbei_4433F Cbei_4433R	AAGCTGCAAAGCAGTCAAATC ACTTAGCTCCAACGTCAATACC	ABC transporter (*mglA*)
Cbei_4307F Cbei_4307R	TCAGATGATGGAGCAGGAATAAA TCCAACTCCTCTTCCTGAGATA	CheA signal transduction histidine kinase (*cheA*)
Cbei_0679F Cbei_0679R	ACAGAGCCGACAACAGAAATA GCAGCTTCTCCTCCTGATAAA	ABC transporter (*yfmM*)

### Aspartic acid supplementation of the fermentation medium

Following the outcome of RNA sequencing (i.e., reduced expression of aspartate-dependent NAD biosynthesis genes), *C. beijerinckii*_mgsA+mgR and *C. beijerinckii*_p459 were grown in aspartic acid-supplemented lactose-based P2 medium as described earlier. Aspartic acid was added at 0 h, and fermentation was conducted for 84 h. All cultures were supplemented with aspartic acid (2 g/L) and erythromycin (15 µg/mL) at 0 h. Samples were collected every 12 h and analyzed for the concentrations of acetone, butanol, ethanol, acetic acid, and butyric acid as previously reported (12) using gas chromatography. Concomitantly, samples were analyzed for pH and OD_600_ according to previously reported protocols (12).

### Fermentation in iron-deficient medium

A large number of genes involved in iron metabolism showed reduced mRNA abundance in *C. beijerinckii*_mgsA+mgR relative to *C. beijerinckii*_p459. Consequently, both strains were cultivated in the same fermentation medium as described earlier (for RNA sequencing). However, to assess the effect of iron on growth and solvent production in both strains, they were grown with a fivefold reduction in the concentration of iron (2.0 mg/L FeSO_4_.7H_2_O in place of 10.0 mg/L). Cultures were grown for 72 h and sampled hourly for growth and acid (acetic and butyric acids) and solvent (acetone, butanol, and ethanol) production.

### Microscopy

*C. beijerinckii*_mgsA+mgR and *C. beijerinckii*_p459 were grown as described for RNA sequencing. After OD_600 nm_ ~ 1.0, triplicate samples (1 mL) were collected for microscopic analysis. A Nikon Eclipse Ti series microscope (Nikon, Melville, NY, USA) with a 100× oil immersion objective lens was used to capture images on a 1% agarose pad. Two microliters of the culture was placed on the agarose pad and allowed to dry for ~5 min. Afterwards, the pads were covered with glass slide and then viewed with oil immersion objective.

## RESULTS

### Summary of transcriptomic profiles of *C. beijerinckii*_mgsA+mgR relative to *C. beijerinckii*_p459

Fold changes in mRNA abundance are presented in Log_2_. A total of 300 and 433 genes showed increases and decreases in mRNA abundance, respectively, in *C. beijerinckii*_mgsA+mgR relative to *C. beijerinckii*_p459 (Table 2). Some of the genes with significantly high mRNA abundance in *C. beijerinckii*_mgsA+mgR include Cbei_3015, Cbei_3778, Cbei_0533, *rpiB*, *crt*, *aroH*, *feoA*, *feoB*, *iolB*, *iolC*, *iolD*, *iolE*, *iolJ*, *CheA*, *fliI*, *CheA*, and *CheB* (Table S1). These genes are involved in electron transfer (e.g., Cbei_3015: flavodoxin), iron uptake, motility and signal transduction, carbohydrate, protein, lipid, or nucleotide metabolism; glycosylation (e.g., Cbei_3778: glycosyl transferase); and DNA recombination (Cbei_0533, recombinase). Genes involved in nutrient/nucleotide transport and metabolism accounted for the most abundant mRNAs (82/27.33%) in *C. beijerinckii*_mgsA+mgR relative to *C. beijerinckii*_p459. Conversely, the mRNAs of genes of unknown function were the least abundant in *C. beijerinckii*_mgsA+mgR relative to *C. beijerinckii*_p459 (166/38.34%; Table 2).

**TABLE 2:** Summary of the up- or down-regulated genes in *C. beijerinckii*_mgsA+mgR versus *C. beijerinckii*_p459

	Genes with increased mRNA abundance		Genes with decreased mRNA abundance	
	No. of genes	Percentage (%)	No. of genes	Percentage (%)
Coenzyme metabolism	5	1.70	7	1.61
Energy production and conservation	22	7.33	29	6.68
Intracellular trafficking and secretion	3	1.00	2	0.46
Secondary structure	0	0.00	5	1.15
Transcription, replication and repair	22	7.33	42	9.70
Translation	6	2.00	6	1.40
Post-translational modification/protein turnover/chaperone functions	0	0.00	6	1.40
Cell motility and signal transduction	52	17.33	15	3.50
Nutrient/nucleotide transport and metabolism	82	27.33	99	22.90
Cell wall/membrane/envelop biogenesis	10	3.33	24	5.53
Lipid biosynthesis and metabolism	14	4.70	10	2.30
Cell cycle control and mitosis	10	3.33	15	3.50
Stress response	2	0.70	8	1.84
Unknown functions	73	24.33	166	38.25
Total genes	300	100	434	100

### Differentially expressed nutrient/nucleotide transport and coenzyme metabolism genes

The more abundant mRNAs in *C. beijerinckii*_mgsA+mgR are largely involved in carbohydrate, amino acid, nucleotide, and iron import and metabolism (Table S1) . Results for various genes are grouped according to gene functions.

#### Lactose and iron uptake and metabolism

Seventeen (20.73%) of the more abundant nutrient/nucleotide transport and metabolism-related mRNAs are associated with the phosphotransferase system (PTS) or the iron acquisition machinery (Table 3). In fact, 23 (28.04%) of the nutrient import and metabolism-related genes with increases in mRNA abundance are involved in carbohydrate import and metabolism, particularly lactose. Similarly, seven (8.54%) of the most abundant mRNAs in this category are involved in iron uptake and metabolism/homeostasis [e.g., hemerythrin-like protein (Cbei_2165) and ferrous iron uptake proteins (three *feoA*s and two *feoB*s)].

**TABLE 3:** Select genes of the phosphotransferase system or iron acquisition machinery with increased mRNA abundance in *C. beijerinckii*_mgsA+mgR relative to *C. beijerinckii*_p459

Gene ID	Gene symbol	Protein ID	Protein function	Fold change (Log_2_)	*P* value
Cbei_4545	–	ABR36654.1	Sugar transporter/general substrate transporter	4.98	0.0000
Cbei_3873	–	ABR35987.1	PTS system mannose/fructose/sorbose family transporter subunit IIB	4.78	0.007564654
Cbei_4560	–	ABR36669.1	PTS system fructose subfamily IIA component	4.36	0.000679059
Cbei_4638	–	ABR36746.1	PTS system lactose/cellobiose family transporter subunit IIC	3.52	0.0000
Cbei_4640	–	ABR36748.1	PTS system lactose/cellobiose-specific transporter subunit IIA	3.32	6.52E−95
Cbei_4197	*feoA*	ABR36307.1	FeoA family protein	3.24	3.71E−16
Cbei_4639	–	ABR36747.1	Phosphotransferase system, lactose/cellobiose-specific IIB subunit	3.22	3.18E*−*38
Cbei_4198	*feoA*	ABR36308.1	FeoA family protein	3.22	0.0008
Cbei_4559	–	ABR36668.1	PTS system sorbose subfamily IIB component	2.78	1.87E*−*12
Cbei_4196	*feoB*	ABR36306.1	Ferrous iron transport protein B	2.65	2.61E−153
Cbei_4557	–	ABR36666.1	PTS system mannose/fructose/sorbose family IID component	2.45	5.21E*−*42
Cbei_4558	–	ABR36667.1	PTS system sorbose-specific transporter subunit IIC	2.34	1.01E*−*32
Cbei_2534	*feoA*	ABR34690.1	FeoA family protein	1.95	6.46E*−*09
Cbei_0336	*srlA*	ABR32524.1	PTS system glucitol/sorbitol-specific transporter subunit IIC	1.67	8.70E*−*33
Cbei_2535	*feoB*	ABR34691.1	Ferrous iron transport protein B	1.66	2.47E*−*84
Cbei_0337	*srlE*	ABR32525.1	PTS system glucitol/sorbitol-specific transporter subunit IIBC	1.60	6.65E*−*48
Cbei_2320	*treP*	ABR34480.1	PTS system trehalose-specific transporter subunit IIBC	1.28	1.86E*−*21

^*a*^ –, not applicable.

#### Coenzyme metabolism

The mRNAs of genes involved in *de novo* biosynthesis of NAD (via L-aspartate) and pyridoxine (vitamin B_6_) were less abundant in *C. beijerinckii*_mgsA+mgR relative to *C. beijerinckii*_p459, whereas genes encoding proteins involved in the biosynthesis of thiamine, cobalamin (vitamin B_12_), and folate (vitamin B_9_, e.g., 4-amino-4-deoxychorismate lyase—*mltG*) showed greater mRNA abundance (Tables S1 and S2). Genes involved in the biosynthesis of branched chain amino acids (BCAs—valine, leucine, and isoleucine), coenzyme A, and pantothenate (vitamin B_5_) exhibited contrasting patterns of expression. For example, while dihydroxy-acid dehydratase (*ilvD*; Cbei_1519) involved in the biosynthesis of BCAs and vitamin B_5_ showed 3.03-fold reduced mRNA abundance, pantetheine-phosphate adenylyltransferase (*coaD*; Cbei_1160;), which takes part in the biosynthesis of coenzyme A, was 1.32-fold more abundant (Tables S1 and S2).

#### Myo-inositol metabolism

The mRNAs of the myo-inositol catabolic operon (*iolB*, *C*, *D*, *E*, and *J*) were particularly abundant in *C. beijerinckii*_mgsA+mgR, with fold increases ranging from 3.15 to 4.49 (Table S1).

#### Biosynthesis of aromatic amino acids

Some genes associated with the biosynthesis of aromatic amino acids (tyrosine, tryptophan, and phenylalanine) were more abundant in *C. beijerinckii*_mgsA+mgR. Examples include phospho-2-dehydro-3-deoxyheptonate aldolase (*aroH*), indole-3-glycerol-phosphate synthase (*trpC*), and anthranilate synthase component I (*trpE*), a key enzyme in the indole/tryptophan biosynthesis pathway (22; Table S1). Both *trpC* and *trpE* lie within the same gene cluster and exhibited similar levels of mRNA abundance.

#### Cysteine and methionine biosynthesis and metabolism

The mRNAs of Cbei_0622 and Cbei_0630, both of which code for cysteine synthase and a cystathionine gamma-synthase gene (*metC*; Cbei_0629), involved in cysteine and methionine biosynthesis were less abundant in *C. beijerinckii*_mgsA+mgR (Table S2). In addition, the mRNAs of cysteine desulfurase genes (*csd*; Cbei_0585 and *csd2*; Cbei_2599) were 2.60- and 1.40-fold less abundant, respectively. Likewise, the O-acetylhomoserine aminocarboxypropyltransferase gene (*metY*; Cbei_3543) involved in cysteine and methionine metabolism showed reduced mRNA abundance.

#### Metabolism/biosynthesis of other amino acids, purines, and pyrimidines

Carbamoylphosphate synthase [small (*carA*) and large (*carB*)] subunits were 1.6- and 1.42-fold more abundant in *C. beijerinckii*_mgsA+mgR, respectively (Table S1). Carbamoylphosphate synthase is a highly conserved enzyme that catalyzes a step in *de novo* synthesis of arginine and pyrimidines (23). Conversely, phosphoribosylformylglycinamidine synthase (*purL*) and 5-aminoimidazole-4-carboxamide ribonucleotide transformylase (*purH*), which partake in purine biosynthesis, underwent reduced mRNA abundance (Table S2). The mRNAs for asparagine synthase (*asnB*), serine-pyruvate transaminase (Cbei_0759, which participates in glycine, serine, and threonine metabolism), reduced considerably. Another noteworthy amino acid-related gene with reduced mRNA abundance is aspartate-semialdehyde dehydrogenase [*asd*, which plays a crucial role in amino acid and metabolite biosynthesis (24)]. Furthermore, the mRNAs of genes involved in the biosynthesis of lysine and the related bacterial diaminopimelate (a precursor of peptidoglycan) in Gram-positive bacteria (25) were particularly less abundant. Notable examples include dihydrodipicolinate synthase (*dapA*), dihydrodipicolinate reductase (*dapB*), diaminopimelate decarboxylase (*lysA*), and cytidyltransferase (*aepX*; (Table S2). Both *dapA* and *dapB* lie within the same gene cluster, whereas *aepX* lies in a different operon alongside nucleotidyl transferase (Cbei_4339), which exhibited approximately the same degree of reduction in mRNA abundance as *aepX*.

#### Phosphate uptake and metabolism

The results showed that the mRNA levels of two copies of *phoU* (Cbei_1131 and Cbei_1132; Table S2) reduced significantly in *C. beijerinckii*_mgsA+mgR. The protein product of *phoU* (PhoU) is a negative regulator of Pho regulon genes under phosphate-replete conditions (26). Concomitantly, *ppk1* (polyphosphate kinase) was likely down-regulated 2.60-fold.

#### Differential expression patterns of cell motility and signal transduction genes

The genes involved in cell motility and signal transduction accounted for the third most abundant category of mRNAs (Table 2; Table S2). Among the cell motility and signal transduction genes with increased mRNA abundance, genes associated with biosynthesis, assembly, and function of the flagellum accounted for 25.00%. Similarly, 25.00% of the more abundant cell motility and signal transduction mRNAs is that of methyltransferases genes involved in the reception, processing, and transmission of signals from chemoreceptors to the flagella motors. Histidine kinases and methyl-accepting chemotaxis sensory transducers (MCPs) accounted for 17.00% of the cell motility and signal transduction genes with increased mRNA abundance. Conversely, only 15 (3.50%) of the genes with decreased mRNA abundance are associated with cell motility and signal transduction, out of which 5 genes code for MCPs (Table 2; Table S2).

#### The mRNA profiles of energy production and conservation genes

The mRNAs of electron transfer FAD-dependent genes were predominantly more abundant in *C. beijerinckii*_mgsA+mgR (12/54.60%; Table S1), including flavodoxin, which showed the greatest increase (5.71-fold) in mRNA abundance relative to *C. beijerinckii*_p459. Notably, 23.81% of the potentially up-regulated genes involved in energy production and conservation encode iron-dependent proteins. Likewise, the results suggest that 13 genes [e.g., dinitrogenase iron-molybdenum cofactor biosynthesis protein (Cbei_0627) and rubredoxin-type Fe(Cys)4 protein (Cbei_1012)] that encode iron-dependent proteins (46.43%) were down-regulated in *C. beijerinckii*_mgsA+mgR (Table S2). Seven of the 13 genes with decreased mRNA abundance that code for iron-dependent proteins associated with energy production and conservation are “iron-intensive” Fe-S (4Fe-4S/2Fe-2S) cluster proteins (Table S2). Examples include Cbei_0795, Cbei_2128 and Cbei_3544, and Cbei_4835 [ferredoxin iron-sulfur binding domain protein (4Fe-4S), ferredoxin-NADP^+^ reductase subunit alpha, and 2 4Fe-4S ferredoxins, respectively].

#### Differential expression of genes involved in transcription, replication, repair and translation

As shown in Table 2, the mRNA levels of genes involved in transcription, replication, repair, and translation (TRRT) were the fourth most up-regulated and third most down-regulated categories, during the growth of *C. beijerinckii*_mgsA+mgR on lactose. Although TRRT genes accounted for similar percentages of the potentially up- (9.33%) and down-regulated (11.10%) genes in *C. beijerinckii*_mgsA+mgR, the actual number of TRRT genes with reduced mRNA abundance (48 genes) was 71.42% higher than that that was more abundant (28 genes). Interestingly, the mRNAs of genes for three periplasmic binding protein/LacI transcriptional regulators (Cbei_4434, Cbei_2377, and Cbei_0334) involved in lactose metabolism were significantly more abundant (Table S1). Similarly, TRRT genes involved in transcriptional regulation of signal transduction (ECF subfamily RNA polymerase sigma-24 factor; Cbei_1302) showed considerable increases in mRNA abundance. The mRNA levels of the iron-dependent transcription repressor (*ideR*; Cbei_4069), which regulates intracellular free iron concentration (27), decreased 3.20-fold. Other notable TRRT genes with increased mRNA abundance include DeoR family transcriptional regulator (Cbei_0466), a repressor of sugar metabolism (28), MarR family transcriptional regulator (Cbei_0302), and nuclease SbcCD subunit D (*sbcD*; Cbei_0980) that facilitates DNA transcription (Table S1). Conversely, MerR family transcriptional regulator (Cbei_2721), BadM/Rrf2 family transcriptional regulator (Cbei_2221), and two copies of glucose-inhibited division protein A gene (*gidA*), involved in growth and stress response, exhibited varying degrees of reduced levels of mRNA abundance (Table S2).

#### The mRNA levels of genes involved cell wall/membrane/envelop biogenesis

The mRNAs of genes involved in the biosynthesis of cell wall and membrane components were less abundant, with a few specific genes involved in peptidoglycan biosynthesis exhibiting greater mRNA abundance. Of note are alanine racemase (*alr*; Cbei_4113), two copies of membrane-bound O-acyl transferase family protein (*algI*; Cbei_1088 and *dltB*; Cbei_4331), D-alanine-D-alanine ligase (*ddl*; Cbei_0581), and undecaprenyl-phosphate galactose phosphotransferase (Cbei_4575), with significant increases in mRNA abundance (Table S1). These genes are involved in the biosynthesis of different components of the bacterial cell wall or membrane architecture and their ancillary structures including D-alanine (D-Ala; ), triacylglycerol, d-alanyl-d-alanine dipeptide (29), and the precursor of O-polysaccharide. Concomitantly, a wide range of genes involved in cell wall/membrane remodeling and biogenesis, capsule formation, and growth appeared to be strongly down-regulated (Table S2). Notable examples include UDP-glucose 6-dehydrogenase (*wbpA1*; Cbei_4708), mannose-1-phosphate guanylyltransferase (*manC*; Cbei_0318), the gene for NLP/P60 protein (Cbei_1242), and UDP-N-acetylglucosamine 1-carboxyvinyltransferase (*murA*; Cbei_0421). Other potentially down-regulated genes associated with cell wall/membrane/capsular structures include glucosamine-fructose-6-phosphate aminotransferase (*glmS*; Cbei_0246) and genes of the L-rhamnose—an important component of cell wall lipopolysaccharide—biosynthesis operon [*rfbA* (Cbei_2602), *rfbB* (Cbei_2578), *rfbC* (Cbei_2601), and *rfbD* (Cbei_2579); (Table S2)].

#### The mRNA profiles of genes associated with lipid/fatty acid metabolism and biosynthesis

Similar numbers of genes associated with lipid/fatty acid metabolism and biosynthesis appeared to be up- (14 genes) and down-regulated (10 genes) in lactose-grown *C. beijerinckii*_mgsA+mgR relative to *C. beijerinckii*_p459 (Tables S1 and S2). The genes 4′-phosphopantetheinyl transferase (Cbei_0687), acyl-ACP thioesterase (Cbei_0691), and thioesterase (Cbei_0681), involved in various stages of fatty acid biosynthesis (30–32), exhibited significant increases in mRNA abundance in *C. beijerinckii*_mgsA+mgR relative to *C. beijerinckii*_p459. Additionally, genes of the *fab* fatty acid chain elongation cluster [beta-ketoacyl synthase (*fabF*; Cbei_1072), beta-hydroxyacyl-(acyl-carrier-protein) dehydratase (*fabZ*; Cbei_1074), and 3-oxoacyl-(acyl-carrier-protein) reductase (*fabG*; Cbei_1071); 33] showed increases in mRNA abundance. On the other hand, the mRNAs of genes that take part in fatty acid metabolism [alpha/beta fold hydrolases (Cbei_3932 and Cbei_3997), 3-oxoacid CoA-transferase subunits (Cbei_2654 and Cbei_2653), and acetyl-CoA acetyltransferase (Cbei_3630)] were less abundant in *C. beijerinckii*_mgsA+mgR relative to *C. beijerinckii*_p459.

### Quantitative RT-PCR

A total of 11 differentially expressed genes were studied by RT-qPCR. Genes were chosen to assess the different degrees of increase/decrease in mRNA abundance (high, low, or medium) observed with RNA sequencing. RT-qPCR results mirrored the increases/decreases in mRNA abundance observed with RNA sequencing. Specifically, Cbei_0792 (quinolinate synthetase—*nadA*), Cbei_0794 (nicotinate-nucleotide pyrophosphorylase/quinolinate phosphoribosyl transferase—*nadC*), Cbei_0793 (L-aspartate oxidase—*nadB*), Cbei_0620 (oxidoreductase/nitrogenase—*nifD1*), Cbei_2281 [oxidoreductase FAD/NAD(P)-binding domain protein], and Cbei_1546 (hypothetical protein) showed comparable decreases in mRNA abundance relative to RNA sequencing (Table 4). Similarly, Cbei_3015 (flavodoxin), Cbei_4307 (CheA signal transduction histidine kinase—*cheA*), Cbei_3778 (glycosyl transferase family protein), and the ABC transporters (Cbei_4433—*mglA* and Cbei_0679—*yfmM*) exhibited increases in mRNA abundance similar to those obtained with RNA sequencing.

**TABLE 4:** qPCR verification of differentially expressed genes in *C. beijerinckii*_mgsA+mgR relative to *C. beijerinckii*_p459^*a*^

Gene ID	Gene name	Fold change (Log_2_)	
		RNA sequencing (*P* value)	RT-qPCR (SD)
Cbei_0792	Quinolinate synthetase (*nadA*)	−7.14 ± 9.1E*−*237	−9.39 ± 0.000
Cbei_0794	Nicotinate-nucleotide pyrophosphorylase/quinolinate phosphoribosyl transferase (*nadC*)	−5.90 ± 0.0000	−6.68 ± 0.003
Cbei_0793	L-Aspartate oxidase (*nadB*)	−5.50 ± 8.71E*−*112	−9.60 ± 0.000
Cbei_0620	Oxidoreductase/nitrogenase (*nifD1*)	−4.42 ± 2.82E*−*192	−1.40 ± 0.060
Cbei_2281	Oxidoreductase FAD/NAD(P)-binding domain protein	−1.91 ± 0.2378	−0.50 ± 0.394
Cbei_1546	Hypothetical protein	−0.999 ± 2.12E*−*35	−0.96 ± 0.431
Cbei_3015	Flavodoxin	5.71 ± 0.0002	6.50 ± 0.308
Cbei_4307	CheA signal transduction histidine kinase (*cheA*)	1.71 ± 9.07E*−*157	4.16 ± 0.160
Cbei_3778	Glycosyl transferase family protein	5.19 ± 0.0014	4.08 ± 0.622
Cbei_4433	ABC transporter related (*mgl*A)	1.90 ± 6.96E*−*80	2.33 ± 0.243
Cbei_0679	ABC transporter (*yfmM*)	1.12 ± 1.31E−55	1.10 ± 0.270

^*a*^ SD, standard deviation (*n* = 3).

### Effect of aspartic acid supplementation on the fermentation profiles of *C. beijerinckii*_mgsA+mgR

**Fig 1:**
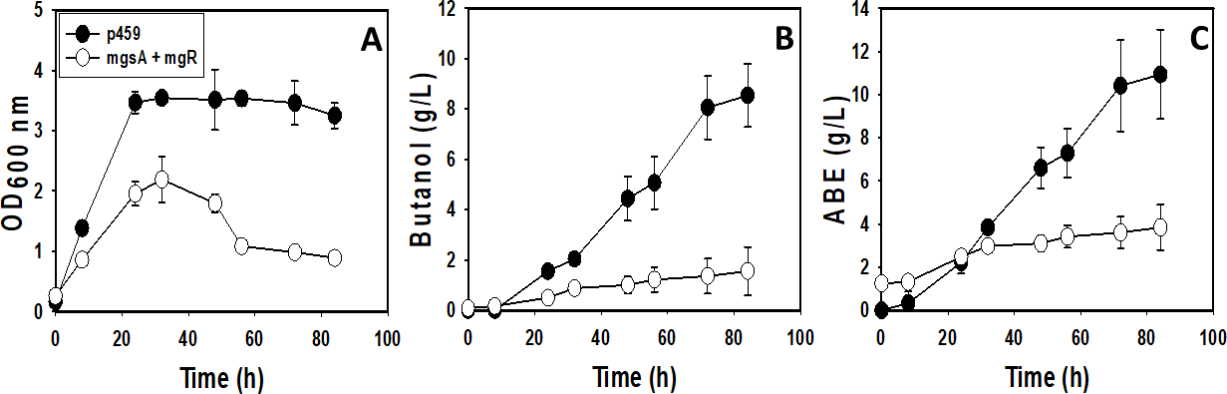
Growth and solvent profiles of *C. beijerinckii*_mgsA+mgR and *C. beijerinckii*_p459 in aspartate-supplemented cultures. (**A**) Growth (OD_600 nm_). (**B**) Butanol concentrations. (**C**) Total ABE concentrations. (*n* = 3).

**Fig 2:**
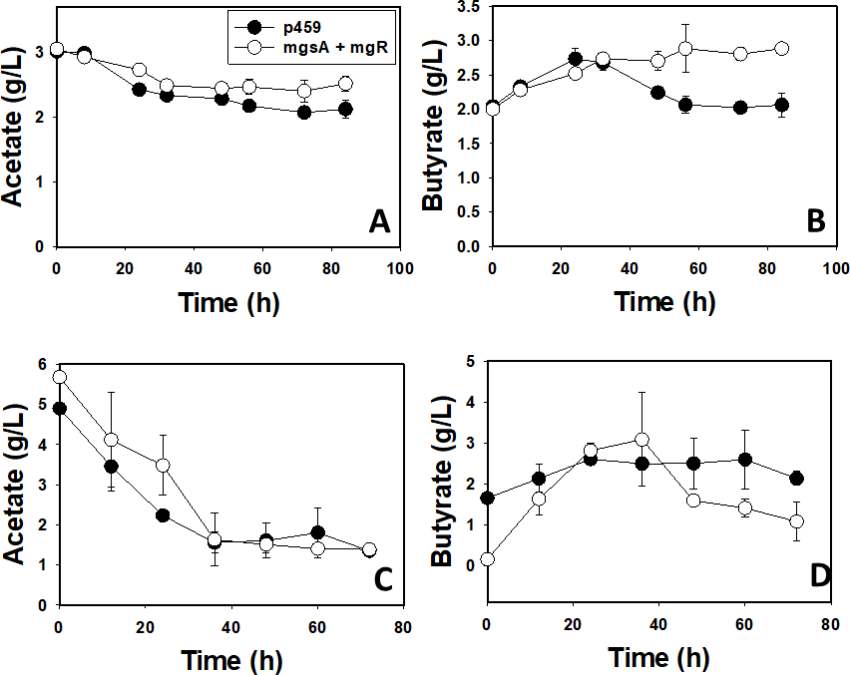
Acetate and butyrate concentrations in aspartate-supplemented and un-supplemented cultures of *C. beijerinckii*_mgsA+mgR and *C. beijerinckii*_p459. (**A**) Acetate concentrations in aspartate-supplemented cultures. (**B**) Butyrate concentrations in aspartate-supplemented cultures. (**C**) Acetate concentrations in aspartate un-supplemented cultures. (**D**) Butyrate concentrations in aspartate un-supplemented cultures (*n* = 3).

We previously observed that *C. beijerinckii*_mgsA+mgR exhibits superior growth (35% higher), butanol, and total solvent [acetone-butanol-ethanol (ABE); up to 87% greater] profiles when compared with the control strain (*C. beijerinckii*_p459; 9). Following the observation of possible strong down-regulation of multiple genes involved in *de novo* biosynthesis of NAD via L-aspartate in *C. beijerinckii*_mgsA+mgR, cultures of *C. beijerinckii*_mgsA+mgR and *C. beijerinckii*_p459 were supplemented with aspartic acid (2 g/L). With aspartic acid supplementation, the OD_600_ of *C. beijerinckii*_mgsA+mgR reduced ~62%, relative to the cultures of *C. beijerinckii*_p459 (Fig. 1A). This is 90% lower than that previously observed for the same strain (*C. beijerinckii*_mgsA+mgR) and only 9% greater than the OD_600_ observed for *C. beijerinckii*_p459 without aspartic acid supplementation (9). Concomitantly, butanol and ABE concentrations decreased 434% and 185%, respectively, in cultures of *C. beijerinckii*_mgsA+mgR relative to those of *C. beijerinckii*_p459 (Fig. 1B and C). These results translate to 235% lower butanol concentration with aspartic acid compared with cultures of *C. beijerinckii*_mgsA+mgR grown without aspartic acid supplementation and 14% greater than that observed for *C. beijerinckii*_p459 in the absence of aspartic acid supplementation (9). Aspartic acid supplementation impaired acid re-assimilation in *C. beijerinckii*_mgsA+mgR but not in *C. beijerinckii*_p459 (Fig. 2). As shown in Fig. 2, addition of aspartic acid to the fermentation medium led to consistently higher concentrations of acetate and butyrate in cultures of *C. beijerinckii*_mgsA+mgR when compared with *C. beijerinckii*_p459 (Fig. 2A and B). Consequently, 19% and 41% more acetate and butyrate, respectively, remained in the cultures of *C. beijerinckii*_mgsA+mgR at the end of fermentation, when compared with the cultures of *C. beijerinckii*_p459. Conversely, in cultures without aspartic acid, both strains exhibited similar patterns of acid re-assimilation, particularly, acetate (Fig. 2C and D). In fact, without aspartic acid supplementation, at the end of fermentation, both *C. beijerinckii*_mgsA+mgR and *C. beijerinckii*_p459 contained 1.39 and 1.35 g/L acetate, respectively, whereas the cultures of *C. beijerinckii*_p459 contained 99% more butyrate (2.13 g/L) than those of *C. beijerinckii*_mgsA+mgR (1.07 g/L).

### Effect of low iron concentration on *C. beijerinckii*_mgsA+mgR relative to *C. beijerinckii*_p459

**Fig 3:**
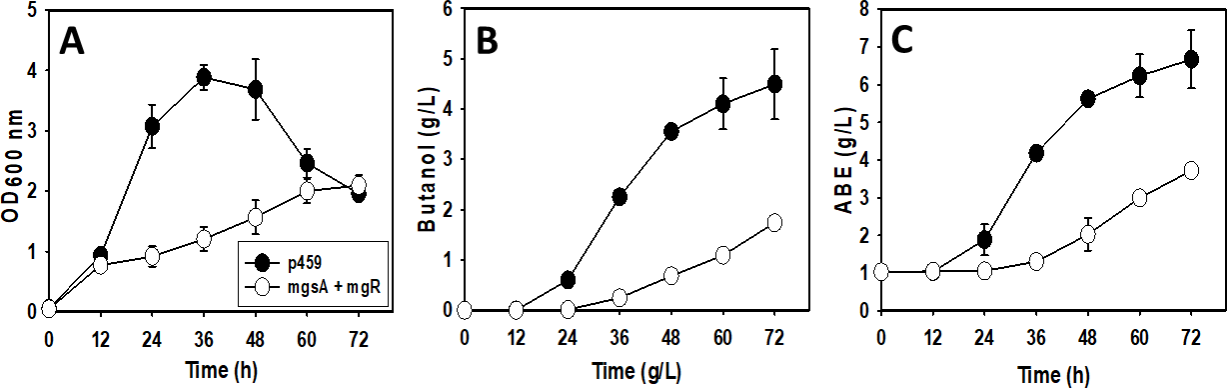
Growth profiles and solvent concentrations in cultures of *C. beijerinckii*_mgsA+mgR and *C. beijerinckii*_p459 grown in iron-limited medium. (A) Optical density (OD_600 nm_). (B) Butanol concentration. (C) ABE concentration (*n* = 3).

In iron-limited medium, *C. beijerinckii*_p459 exhibited 86% higher maximum OD_600_ than *C. beijerinckii*_mgsA+mgR (Fig. 3A). Likewise, butanol and ABE concentrations increased 159% and 80%, respectively, in cultures of *C. beijerinckii*_p459 relative to *C. beijerinckii*_mgsA+mgR (Fig. 3B and C). Under iron limiting condition, the maximum OD_600_ and butanol and ABE concentrations of *C. beijerinckii*_p459 were 1.03% more, 19% less, and 6.62% more, respectively, when compared with *C. beijerinckii*_mgsA+mgR grown on lactose with standard (10 mg/L) iron concentration (9).

### Morphological features of *C. beijerinckii*_mgsA+mgR and *C. beijerinckii*_p459

**Fig 4:**
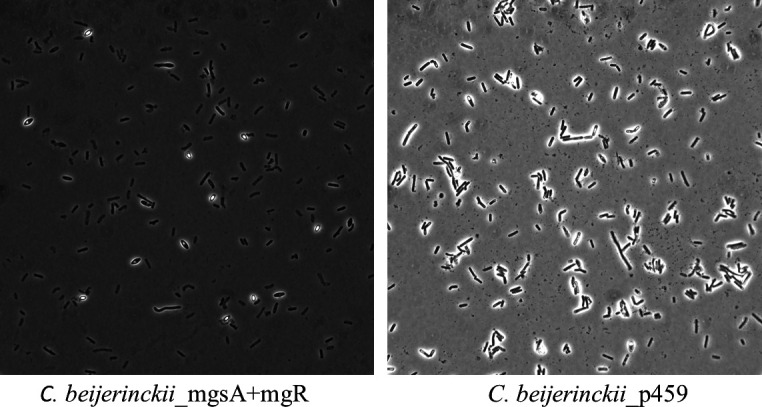
Morphological features and distribution of the cells of *C. beijerinckii*_mgsA+mgR and *C. beijerinckii*_p459 grown on lactose for 24 h.

Examination of *C. beijerinckii*_mgsA+mgR and *C. beijerinckii*_p459 using a Nikon Eclipse Ti series microscope revealed that while cells of *C. beijerinckii*_mgsA+mgR appeared predominantly as discrete, individual cells, *C. beijerinckii*_p459 occurred largely in clusters of closely attached cells (Fig. 4).

## DISCUSSION

We previously observed improved growth and butanol production by *C. beijerinckii*_mgsA+mgR (relative to the empty plasmid control strain: *C. beijerinckii*_p459; 9). Here, we present the global transcriptomic profile of *C. beijerinckii*_mgsA+mgR relative to *C. beijerinckii*_p459, in an effort to delineate the underlying molecular basis for enhanced growth and solvent production by lactose-grown *C. beijerinckii*_mgsA+mgR. Broadly, when compared with *C. beijerinckii*_p459, *de novo* biosynthesis of NAD via L-aspartate, vitamin B_6_, purines, and capsular exopolysaccharides were likely down-regulated in *C. beijerinckii*_mgsA+mgR. Similarly, asparagine and lysine biosynthesis; glycine, serine, and threonine metabolism; and cysteine and methionine biosynthesis and metabolic pathways appeared to be down-regulated in *C. beijerinckii*_mgsA+mgR. On the other hand, the mRNA profiles suggest that phosphate, iron, purine, pyrimidine, and peptide uptake were up-regulated in *C. beijerinckii*_mgsA+mgR. In addition, the mRNA profiles indicate that the two-component signal transduction system and cell motility, flavodoxin, and flavoproteins were up-regulated in *C. beijerinckii*_mgsA+mgR, relative to *C. beijerinckii*_p459. The results are discussed in detail under select functional categories.

### Possible interaction between methylglyoxal-mediated protein glycation and intracellular ferrous iron concentration

The transcriptomic repertoire of lactose-grown *C. beijerinckii*_mgsA+mgR suggests that the cells might experience lower levels of intracellular ferrous iron, which likely triggered a series of changes in gene expression patterns that led to enhanced growth, lactose utilization, and solvent production. Ferrous iron plays a vital role in ABE fermentation. Above 10 mg/L, increasing ferrous iron concentration impairs growth, sugar utilization, and solvent production, with concomitant acid accumulation in solvent-producing *Clostridium* species (34). Conversely, lower concentrations of iron favor sugar utilization, growth, and ABE production (34). Notably, *C. beijerinckii*_mgsA+mgR consumed 183.30% more lactose than *C. beijerinckii*_p459 (9). It is important to highlight that both *C. beijerinckii*_mgsA+mgR and *C. beijerinckii*_p459 were grown in the same medium containing 10 mg/L FeSO_4_.7H_2_O. However, the expression profiles of iron regulatory and uptake genes in *C. beijerinckii*_mgsA+mgR clearly suggest likely concerted cellular effort to increase iron uptake in *C. beijerinckii*_mgsA+mgR. For example, IdeR is an iron-responsive transcription factor that functions as the gatekeeper of iron uptake (27). Interestingly, *ideR* was the second most down-regulated transcription factor in *C. beijerinckii*_mgsA+mgR. IdeR controls the expression of several genes involved in iron uptake, metabolism, and storage. Significant reduction in the mRNA transcripts of *ideR* is indicative of possible lower intracellular levels of ferrous iron in *C. beijerinckii*_mgsA+mgR, which might explain increased expression of the iron uptake genes *feoA* (three copies) and *feoB*.

**Fig 5:**
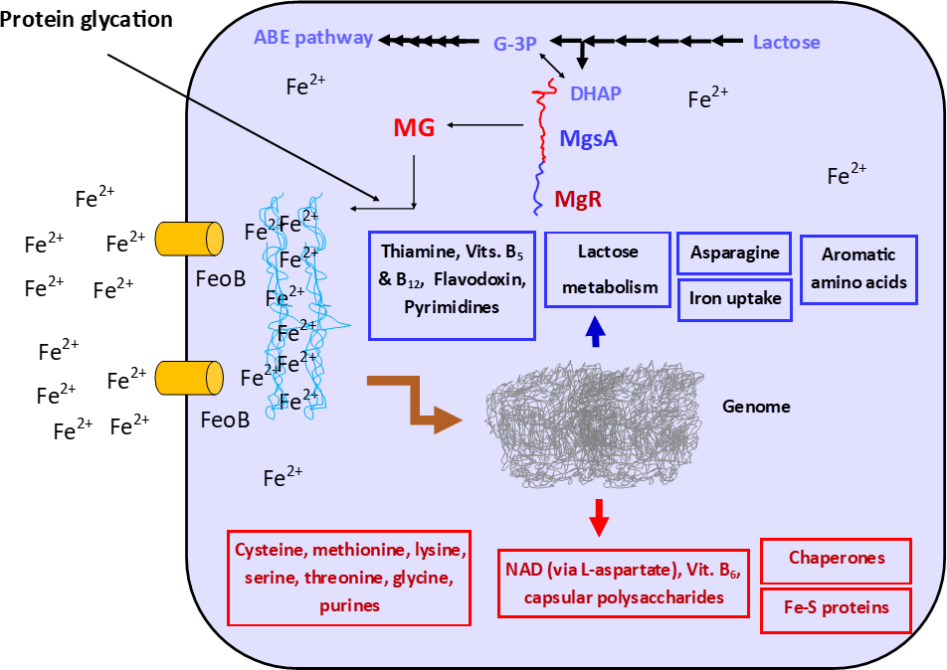
Summary of gene expression profiles in lactose-grown *C. beijerinckii*_mgsA+mgR relative to *C. beijerinckii*_p459. MgsA (methylglyoxal synthase) represents the protein product of *mgsA* fused with MgR. This is the recombinant pathway responsible for likely methylglyoxal production. Blue panels and arrows denote possible up-regulated functions, whereas red arrows and panels denote functions that were likely down-regulated. MG-mediated protein glycation can bind Fe^2+^, which may account for increased expression of Fe^2+^ uptake proteins and flavodoxins. Reduced expression of Fe-S proteins including key players in the NAD *de novo* biosynthesis pathway via L-aspartate likely triggered tryptophan (aromatic amino acid) biosynthesis, as an alternative route for NAD production. Reduced availability of Fe^2+^ presumably accounts for enhanced growth and lactose utilization. G-3P, glyceraldehyde-3-phosphate; DHAP, dihydroxyacetone phosphate.

Methylglyoxal (MG), the product of the MgsA-catalyzed reaction, is a highly reactive α-oxoaldehyde that exerts severe stress on biological cells, which results in complex formation with proteins via glycation (35–37). Iron has been reported to promote MG-mediated protein damage (36). Therefore, we speculate that the MG product of MgsA likely interacts with ferrous iron and Fe-S proteins, which are abundant in *C. beijerinckii* (38). The resulting iron binding MG-protein complexes likely make free iron less available within the cell (Fig. 5). This assertion is consistent with the collective expression profiles of cysteine desulfurase (*csd*), cysteine and methionine biosynthesis and metabolic enzymes, iron import permeases (*feoA* and *feoB*), hemerythrin, *ideR*, flavodoxin, and other flavoproteins (Fig. 5). Flavodoxins are electron-transfer proteins that participate in a wide variety cellular reactions (39). Notably, flavodoxins are prevalent in cells growing in iron-limited environments (39–41), and the flavodoxin mRNA was the most abundant in *C. beijerinckii*_mgsA+mgR (Table S1). In addition, hemerythrin participates in iron homeostasis and has been reported to repair iron centers in *Escherichia coli* (42).

Whereas three distinct Fe-S cluster assembly machineries have been characterized in bacteria, irrespective of the assembly type, cysteine desulfurase (Csd) mediates the assembly of transient clusters on scaffold proteins and consequent relocation of pre-formed clusters to apo-proteins (43). It is plausible therefore that down-regulation of Fe-S proteins and limited intracellular iron concentration within the cell elicited attendant down-regulation of *csd* and, ultimately, Fe-S cluster biosynthesis and assembly. This is possibly linked to down-regulation of cysteine and methionine (sulfur bearing amino acids) uptake and metabolism, of which cysteine plays an important role in the anchoring Fe-S clusters onto designated proteins (43). The growth kinetics and solvent titers of *C. beijerinckii*_mgsA+mgR and *C. beijerinckii*_p459 grown in iron-limited medium support this assumption (Fig. 3). Reduced growth and solvent production in *C. beijerinckii*_mgsA+mgR in an iron-limited medium may be ascribed to severe intracellular iron shortage, which may have resulted in reduced growth and solvent biosynthesis. Conversely, in the absence of MG-mediated iron complexation in *C. beijerinckii*_p459, limiting iron concentration enhanced growth and butanol production.

### The contrasting morphologies of *C. beijerinckii*_mgsA+mgR and *C. beijerinckii*_p459 and expression patterns of signal transduction and motility genes

Enhanced growth and cell viability can manifest in the form of improved cell motility and viability. Thus, the expression patterns of two-component signal transduction and cell motility genes in *C. beijerinckii*_mgsA+mgR clearly indicate probable overall greater cell viability when compared with *C. beijerinckii*_p459. This is in agreement with the reduced mRNA abundance observed for genes involved the biosynthesis of capsular polysaccharides in *C. beijerinckii*_mgsA+mgR, which further indicates more active, discrete, and motile cells. This is because, capsular polysaccharides mediate biofilm formation and adherence of bacterial cells to surfaces (44). Indeed, microscopic examination of the two strains clearly accentuates this notion, as cells of *C. beijerinckii*_mgsA+mgR were observed to be more discrete than those of *C. beijerinckii*_p459, which were largely clustered (Fig. 4). More discrete and active cells suggest overall more robust growth and solvent production, as observed with *C. beijerinckii*_mgsA+mgR in comparison to *C. beijerinckii*_p459 (9).

### NAD and butanol biosynthesis

Despite its pivotal role under fermentative condition, *de novo* biosynthesis of NAD via L-aspartate appeared to be down-regulated in *C. beijerinckii*_mgsA+mgR. The protein product of *nadA* (quinolinate synthetase), which catalyzes a key step in the NAD *de novo* biosynthesis pathway via L-aspartate, is an Fe-S protein. Given substantial down-regulation of Fe-S proteins in *C. beijerinckii*_mgsA+mgR, it is plausible that the expression of *nadA* was depressed as a result of this broader trend. *C. beijerinckii*_mgsA+mgR appears to exhibit a remodeled metabolic profile that circumvents the need for NAD biosynthesis via L-aspartate. For instance, NADH dehydrogenase (Cbei_4112), which re-oxidizes NADH, was more abundant in *C. beijerinckii*_mgsA+mgR. At the time of sampling for RNA extraction, butanol concentrations—an outlet for NADH re-oxidation—were similar between *C. beijerinckii*_mgsA+mgR and *C. beijerinckii*_p459 (9). Concomitantly, Fe-only hydrogenase (Cbei_4110), which is also involved in NADH re-oxidation, exhibited increased mRNA abundance in *C. beijerinckii*_mgsA+mgR. The activities of the enzyme products of both genes likely allow *C. beijerinckii*_mgsA+mgR to maximize available NAD via re-oxidation. In fact, the growth and solvent profiles of *C. beijerinckii*_mgsA+mgR relative to *C. beijerinckii*_p459 suggest that the prevailing metabolic machinery of the former is more efficient. Thus, addition of 2 g/L L-aspartate likely up-regulated the L-aspartate end of NAD *de novo* biosynthesis, possibly at the expense of another salient nexus in the metabolic network, which in turn, disrupted the metabolic sequence in this strain. This may account for the severely diminished growth, ABE production, and acid re-assimilation observed in aspartic acid-supplemented cultures of *C. beijerinckii*_mgsA+mgR (Fig. 1).

As part of a possibly remodeled metabolism, the mRNA profiles suggest that *C. beijerinckii*_mgsA+mgR likely deploys the tryptophan route to meet/augment cellular NAD requirements. This assumption is supported by the increased mRNA levels observed for *aroH*, *trpC*, and *trpE* in *C. beijerinckii*_mgsA+mgR relative to *C. beijerinckii*_p459. The protein product of *aroH* (phospho-2-dehydro-3-deoxyheptonate aldolase) catalyzes the first of seven steps leading to biosynthesis of chorismate, a precursor of all three aromatic amino acids, *p*-amino butyric acid, folate cofactors, ubiquinone, and menaquinone (45, 46). Concomitantly, only indole-3-glycerol-phosphate synthase (*trpC*) and anthranilate synthase component I (*trpE*), both of which are involved in tryptophan biosynthesis, showed increases in mRNA abundance in this pathway. This indicates that tryptophan may be the major biosynthesis target in the aromatic amino acid pathway. Aside from aspartate, tryptophan is a precursor of quinolinate in an alternative arm of the *de novo* NAD biosynthesis pathway. Thus, we speculate that increased expression of tryptophan biosynthesis genes might represent an alternative route for NAD production in *C. beijerinckii*_mgsA+mgR.

### Other aspects highlighted by the transcriptomic results

RNA sequencing data showed increased mRNA abundance for the myo-inositol catabolic operon (*iolB*, *C*, *D*, *E*, and *J*) in *C. beijerinckii*_mgsA+mgR. The underlying reason for this is not clear. However, given that the entire operon appeared to be up-regulated, we speculate that the observed increases in mRNA abundance might be due to a cascade effect. Inactivation of the operon or select genes within the operon in *C. beijerinckii*_mgsA+mgR followed by assessing growth and butanol profiles promises to provide more insight into the basis for this result. Similarly, the expression patterns observed for the genes involved in asparagine, lysine, and pyrimidine biosynthesis and threonine and glycine metabolism deserve further attention, to better understand the behavior of lactose-grown *C. beijerinckii*_mgsA+mgR. Furthermore, decreases in mRNA abundance observed for two copies of *phoU* support the possibility of concerted phosphate uptake in *C. beijerinckii_*mgsA+mgR. The reason for this remains unclear and thus warrants additional study.

We are exploring isotope labeling/metabolomics and targeted gene knockouts to better characterize and confirm the roles that some of the genes discussed in this study might play in lactose-grown *C. beijerinckii_*mgsA+mgR. We expect that the results will provide additional insights leading to metabolic engineering of a robust strain capable of efficient biosynthesis of 1,2-PD via the MG bypass. Also, the results will likely advance the engineering of *C. beijerinckii* for improved butanol production. Based on the observed mRNA profiles, *csd*, *nadA*, and *ideR* represent potential metabolic engineering targets, with a view to increasing butanol production in *C. beijerinckii*.

### Conclusion

In this study, we sought to understand the basis for enhanced butanol production by *C. beijerinckii*_mgsA+mgR, a recombinant strain of *C. beijerinckii* heterologously co-expressing *mgsA* and *mgR* from *C. pasteurianum*. The mRNA profiles suggest that *C. beijerinckii*_mgsA+mgR likely recruits the tryptophan route over the L-aspartate route for NAD production. In parallel, genes involved in iron import exhibited greater mRNA preponderance, alongside reduced mRNA abundance for a large number of genes encoding Fe-S proteins. This is indicative of likely intracellular ferrous iron limitation, possibly due to ferrous iron complexing with methylglyoxal-glycated proteins. While the results presented in this study highlight some potential engineering targets for improving butanol production in *C. beijerinckii*, it is important to mention that mRNA profiles do not always align with actual protein profiles within the cell. Furthermore, not all the genes highlighted in this study have been characterized biochemically following annotation. Therefore, further investigation of the mRNA profiles reported here by knocking out or overexpressing select up-/down-regulated genes will prove instructive. Such a study promises to provide more detailed insights that will better explain the behavior of *C. beijerinckii*_mgsA+mgR grown on lactose, in relation to cell morphology, growth profile, and indeed butanol and ABE concentrations.

## Data Availability

Raw RNA sequencing data will be made available upon request.

## SUPPLEMENTAL MATERIAL

Table S1 aem.01012-24-s0001.pdf Complete list of upregulated genes.

Table S2 aem.01012-24-s0002.pdf Complete list of downregulated genes.

ASM does not own the copyrights to Supplemental Material that may be linked to, or accessed through, an article. The authors have granted ASM a non-exclusive, world-wide license to publish the Supplemental Material files. Please contact the corresponding author directly for reuse.
