# Supplementary material for: Transcriptomic characterization of recombinant Clostridium beijerinckii NCIMB 8052 expressing methylglyoxal synthase and glyoxal reductase from Clostridium pasteurianum ATCC 6013
Source: Appl Environ Microbiol. 2024 Sep 11;90(10):e01012-24. doi: 10.1128/aem.01012-24 (PMC11497831; doi:10.1128/aem.01012-24)
Supplement: Table S1 — Complete list of upregulated genes. [file aem.01012-24-s0001.pdf]

Table S1 Genes upregulated in *C. beijerinckii*_mgsA + mgR relative to *C. beijerinckii*_p459.

Gene ID	Gene symbol	Protein ID	Protein function	Fold change (Log_2)	*p* value
Coenzyme metabolism					
Cbei_0675	-	ABR32861.1	Coenzyme F_{390} synthetase-like protein	1.92	4.18E-118
Cbei_3572	-	ABR35694.1	ApbE family thiamine biosynthesis lipoprotein	1.41	4.31E-07
Cbei_1268	*cobD*	ABR33448.1	Adenosylcobinamide-phosphate synthase CbiB	1.33	3.90E-07
Cbei_1160	*coaD*	ABR33342.1	Pantetheine-phosphate adenylyltransferase	1.32	4.70E-07
Cbei_1989	*moaA*	ABR34159.1	Molybdenum cofactor biosynthesis protein A	1.24	1.81E-11
Energy production and conservation					
Cbei_3015	-	ABR35154.1	Flavodoxin	5.71	0.000156458
Cbei_0311	*etfA2*	ABR32499.1	Electron transfer flavoprotein subunit alpha-like protein	2.76	1.48E-62
Cbei_4585	-	ABR36693.1	NADPH-dependent FMN reductase	2.55	2.82E-114
Cbei_2193	-	ABR34360.1	D-isomer specific 2-hydroxyacid dehydrogenase NAD-binding subunit	2.38	3.48E-06
Cbei_0312	*glcD1*	ABR32500.1	FAD-linked oxidase domain protein	2.27	1.19E-68
Cbei_2990	*ndhG*	ABR35129.1	NADH-ubiquinone/plastoquinone oxidoreductase subunit 6	2.13	0.033116107
Cbei_3014	*hcp*	ABR35153.1	Hydroxylamine reductase	2.20	5.48E-113
Cbei_4507	-	ABR36616.1	FAD-dependent pyridine nucleotide-disulphide oxidoreductase	1.84	8.06E-176
Cbei_4552	*gbsB*	ABR36661.1	Iron-containing alcohol dehydrogenase	1.84	4.39E-52
Cbei_0674	-	ABR32860.1	NAD-dependent aldehyde dehydrogenase-like protein	1.82	1.46E-146
Cbei_3571	-	ABR35693.1	FMN-binding domain-containing protein	1.72	2.96E-11
Cbei_3122	*rbsC*	ABR35258.1	Inner-membrane translocator	1.55	7.21E-64
Cbei_4008	-	ABR36118.1	Malate dehydrogenase	1.54	7.52E-06
Cbei_3570	-	ABR35692.1	FMN-binding domain protein	1.51	1.01E-08
Cbei_0310	*etfB1*	ABR32498.1	Electron transfer flavoprotein, alpha/beta-subunit-like protein	1.40	3.23E-06
Cbei_4112	-	ABR36222.1	NADH dehydrogenase (ubiquinone), 24 kDa subunit	1.34	0.000826776
Cbei_1416	*rbr3A*	ABR33594.1	Rubrerythrin	1.30	3.32E-90
Cbei_2194	-	ABR34361.1	Iron-containing alcohol dehydrogenase	1.28	1.67E-05
Cbei_4110	-	ABR36220.1	Hydrogenase, Fe-only	1.25	9.16E-11
Cbei_4508	*glpA*	ABR36617.1	FAD-dependent oxidoreductase (glycerol-3-phosphate dehydrogenase)	1.20	2.95E-31

Cbei_5054	*cooS*	ABR37160.1	Carbon-monoxide dehydrogenase, catalytic subunit	1.20	5.17E-40
Intracellular trafficking and secretion					
Cbei_0304	-	ABR32492.1	Small multidrug resistance protein	2.28	6.60E-08
Cbei_3879	-	ABR35993.1	Binding-protein-dependent transport system inner membrane protein	1.43	1.25E-17
Cbei_3996	*lepB2*	ABR36106.1	Signal peptidase I	1.20	0.003378634
Transcription, replication and repair					
Cbei_0533	-	ABR32721.1	Recombinase	4.98	0.000551776
Cbei_1302	-	ABR33482.1	ECF subfamily RNA polymerase sigma-24 factor	3.93	1.27E-16
Cbei_3034	-	ABR35173.1	Response regulator receiver protein	1.73	3.47E-69
Cbei_0335	-	ABR32523.1	Glucitol operon activator	1.73	2.77E-14
Cbei_4578	-	ABR36687.1	Accessory gene regulator protein	1.72	1.64E-69
Cbei_2936	-	ABR35078.1	GCN5 N-acetyltransferase	1.68	1.86E-58
Cbei_5053	-	ABR37159.1	BadM/Rrf2 family transcriptional regulator	1.62	5.47E-49
Cbei_0269	-	ABR32458.1	Accessory gene regulator B	1.62	5.17E-46
Cbei_4078	-	ABR36188.1	Periplasmic binding protein/LacI transcriptional regulator	1.97	5.78E-21
Cbei_4434	-	ABR36543.1	Periplasmic binding protein/LacI transcriptional regulator	1.62	1.03E-108
Cbei_2377	-	ABR34537.1	Periplasmic binding protein/LacI transcriptional regulator	1.53	0.00487131
Cbei_0334	-	ABR32522.1	Transcriptional antiterminator BglG	1.51	9.33E-52
Cbei_4819	-	ABR36926.1	Response regulator receiver protein	1.48	0.012414789
Cbei_0888	-	ABR33072.1	Prophage LambdaCh01, transcriptional regulator	1.47	0.018027304
Cbei_0595	*rpoN*	ABR32782.1	RNA polymerase factor sigma-54	1.43	7.62E-67
Cbei_0466	-	ABR32654.1	DeoR family transcriptional regulator	1.37	1.08E-21
Cbei_4367	-	ABR36477.1	DNA ligase-like protein	1.35	0.000228697
Cbei_2355	*xth*	ABR34515.1	Exodeoxyribonuclease III Xth	1.30	2.97E-08
Cbei_0097	*spoIIE*	ABR32287.1	Sporulation stage II, protein E	1.25	9.41E-18
Cbei_0980	*sbcD*	ABR33164.1	Nuclease SbcCD subunit D	1.23	0.005066406
Cbei_4676	-	ABR36784.1	Response regulator receiver protein	1.20	1.15E-12
Cbei_0479	-	ABR32667.1	Exonuclease	1.20	3.01E-29
Translation					
Cbei_5104	*rpmH*	ABR37210.1	Ribosomal protein L34	2.11	0.044657885
Cbei_1782	*valS*	ABR33953.1	valyl-tRNA synthetase	2.01	1.56E-179
Cbei_0092	*hslR*	ABR32282.1	RNA-binding S4 domain protein	1.94	1.61E-07

Cbei_0302	-	ABR32490.1	MarR family transcriptional regulator	1.92	0.039685862
Cbei_4897	*fusA2*	ABR37003.1	Translation elongation factor G	1.45	5.17E-39
Cbei_1104	*alaS*	ABR33286.1	Alanyl-tRNA synthetase	1.38	2.58E-101
Cell motility and signal transduction					
Cbei_4824	-	ABR36931.1	Response regulator receiver protein (chemotaxis protein cheY)	3.32	0.001193013
Cbei_4829	*cheA*	ABR36936.1	CheA signal transduction histidine kinase	3.30	3.24E-118
Cbei_4821	-	ABR36928.1	Methyl-accepting chemotaxis sensory transducer	3.18	3.23E-84
Cbei_4823	-	ABR36930.1	Methyl-accepting chemotaxis sensory transducer	3.17	8.76E-95
Cbei_4828	-	ABR36935.1	Methyl-accepting chemotaxis sensory transducer	2.98	5.02E-113
Cbei_4820	-	ABR36927.1	Response regulator receiver sensor signal transduction histidine kinase	2.97	5.60E-08
Cbei_4822	-	ABR36929.1	CheW protein	2.92	1.20E-14
Cbei_4826	*cheB*	ABR36933.1	Response regulator receiver modulated CheB methylesterase	2.92	1.91E-162
Cbei_2726	-	ABR34877.1	Response regulator receiver protein	2.92	2.56E-62
Cbei_4017	-	ABR36127.1	Methyl-accepting chemotaxis sensory transducer	2.77	4.08E-62
Cbei_4827	*cheR*	ABR36934.1	Protein-glutamate O-methyltransferase/MCP methyltransferase, CheR-type	2.76	1.48E-62
Cbei_2725	-	ABR34876.1	Response regulator receiver sensor signal transduction histidine kinase	2.73	4.31E-58
Cbei_4018	*cheW1*	ABR36128.1	CheW protein	2.62	0.000139504
Cbei_2728	-	ABR34879.1	PAS/PAC sensor signal transduction histidine kinase	2.53	1.84E-19
Cbei_4268	*fliF*	ABR36378.1	Flagellar M-ring protein (FliF)	2.52	3.13E-116
Cbei_2727	-	ABR34878.1	Signal transduction protein	2.40	3.59E-43
Cbei_4272	*motB*	ABR36382.1	OmpA/MotB domain protein	2.26	1.71E-24
Cbei_0875	-	ABR33059.1	Methyl-accepting chemotaxis sensory transducer	2.16	9.94E-145
Cbei_4473	-	ABR36582.1	Metal dependent phosphohydrolase	2.15	0.004918732
Cbei_4265	*fliI*	ABR36375.1	Flagellar protein export ATPase FliI	2.12	4.26E-138
Cbei_5015	-	ABR37121.1	StbA family protein	1.94	1.12E-40
Cbei_4019	*cheA*	ABR36129.1	CheA signal transduction histidine kinase	1.92	6.65E-10
Cbei_4252	*flhF*	ABR36362.1	Flagellar biosynthesis regulator FlhF	1.81	4.98E-61
Cbei_0804	-	ABR32988.1	Methyl-accepting chemotaxis sensory transducer	1.78	1.56E-31
Cbei_4307	*cheA*	ABR36417.1	CheA signal transduction histidine kinase	1.71	9.07E-157

Cbei_4266	*fliH*	ABR36376.1	Flagellar biosynthesis/type III secretory pathway protein-like protein	1.61	1.75E-46
Cbei_4254	*flhB*	ABR36364.1	Bifunctional flagellar biosynthesis protein FliR/FlhB	1.58	3.12E-78
Cbei_2787	-	ABR34937.1	Methyl-accepting chemotaxis sensory transducer	1.52	7.16E-06
Cbei_4020	*cheY2*	ABR36130.1	Response regulator receiver protein	1.51	5.54E-05
Cbei_0680	-	ABR32866.1	O-methyltransferase family protein	1.50	3.60E-77
Cbei_4077	-	ABR36187.1	Methyl-accepting chemotaxis sensory transducer	1.49	2.64E-20
Cbei_4269	*fliE*	ABR36379.1	Flagellar hook-basal body complex subunit FliE	1.48	0.00110445
Cbei_4183	*cheA3*	ABR36293.1	CheA signal transduction histidine kinase	1.48	2.79E-07
Cbei_4253	*flhA*	ABR36363.1	Flagellar biosynthesis protein FlhA	1.46	4.26E-73
Cbei_3471	-	ABR35596.1	Signal transduction protein	1.46	7.26E-24
Cbei_4273	*motA*	ABR36383.1	MotA/TolQ/ExbB proton channel	1.45	4.10E-13
Cbei_0744	-	ABR32928.1	Diguanylate cyclase with PAS/PAC sensor	1.40	8.80E-31
Cbei_3469	-	ABR35594.1	Histidine kinase	1.39	6.20E-09
Cbei_4271	*flgB*	ABR36381.1	Flagellar basal-body rod protein FlgB	1.38	1.11E-07
Cbei_4270	*flgC*	ABR36380.1	Flagellar basal-body rod protein FlgC	1.36	1.64E-13
Cbei_4303	*fliM*	ABR36413.1	Flagellar motor switch protein FliM	1.36	2.52E-113
Cbei_4682	-	ABR36790.1	Methyl-accepting chemotaxis sensory transducer	1.35	4.94E-28
Cbei_3968	-	ABR36078.1	TPR repeat-containing protein	1.34	5.27E-08
Cbei_4291	*fliD*	ABR36401.1	Flagellar hook-associated 2 domain protein	1.35	1.68E-67
Cbei_4267	*fliG*	ABR36377.1	Flagellar motor switch protein FliG	1.28	3.93E-43
Cbei_3923	-	ABR36035.1	Integral membrane sensor signal transduction histidine kinase	1.28	7.82E-66
Cbei_4215	*pilM*	ABR36325.1	Type IV pilus assembly protein PilM	1.27	1.95E-09
Cbei_4015	-	ABR36125.1	Response regulator receiver protein	1.25	0.000870739
Cbei_4016	-	ABR36126.1	Histidine kinase	1.22	3.60E-08
Cbei_0669	-	ABR32856.1	PAS/PAC sensor protein	1.20	3.21E-20
Cbei_3404	-	ABR35530.1	Response regulator receiver protein	1.20	6.60E-07
Cbei_2953	-	ABR35094.1	Methyl-accepting chemotaxis sensory transducer	1.20	1.87E-09
Nutrient/nucleotide transport and metabolism					
Cbei_4545	-	ABR36654.1	Sugar transporter/general substrate transporter	4.98	0.0000

Cbei_3873	-	ABR35987.1	PTS system mannose/fructose/sorbose family transporter subunit IIB	4.78	0.007564654
Cbei_4546	*iolE*	ABR36655.1	Xylose isomerase domain-containing protein (myo-inositol catabolism protein)	4.49	5.48E-244
Cbei_4560	-	ABR36669.1	PTS system fructose subfamily IIA component	4.36	0.000679059
Cbei_4547	-	ABR36656.1	Oxidoreductase domain protein/ myo-inositol 2-dehydrogenase	3.65	1.01E-100
Cbei_4062	-	ABR36172.1	PfpI family intracellular peptidase	3.54	0.000609762
Cbei_4638	-	ABR36746.1	PTS system lactose/cellobiose family transporter subunit IIC	3.52	0.0000
Cbei_4549	*iolB*	ABR36658.1	Myo-inositol catabolism IolB domain protein	3.42	9.78E-161
Cbei_4640	-	ABR36748.1	PTS system lactose/cellobiose-specific transporter subunit IIA	3.32	6.52E-95
Cbei_2384	*xylB*	ABR34544.1	Xylulokinase	3.28	2.07E-98
Cbei_4550	*iolC*	ABR36659.1	Ribokinase-like domain-containing protein	3.26	2.06E-54
Cbei_4548	*iolD*	ABR36657.1	Thiamine pyrophosphate protein, central region	3.25	2.24E-169
Cbei_4197	*feoA*	ABR36307.1	FeoA family protein	3.24	3.71E-16
Cbei_4639	-	ABR36747.1	Phosphotransferase system, lactose/cellobiose-specific IIB subunit	3.22	3.18E-38
Cbei_4198	*feoA*	ABR36308.1	FeoA family protein	3.22	0.000780644
Cbei_1383	-	ABR33562.1	Symporter YidK	3.19	6.78E-199
Cbei_4551	*iolJ*	ABR36660.1	Fructose-bisphosphate aldolase	3.15	1.45E-16
Cbei_4559	-	ABR36668.1	PTS system sorbose subfamily IIB component	2.78	1.87E-12
Cbei_4196	*feoB*	ABR36306.1	Ferrous iron transport protein B	2.65	2.61E-153
Cbei_4584		ABR36692.1	ABC transporter	2.64	3.09E-75
Cbei_4582	-	ABR36690.1	ABC transporter	2.72	8.56E-77
Cbei_2321	*treC*	ABR34481.1	Alpha, alpha-phosphotrehalase	2.59	1.51E-97
Cbei_0685	-	ABR32871.1	Alcohol dehydrogenase GroES domain protein	2.50	2.90E-259
Cbei_4557	-	ABR36666.1	PTS system mannose/fructose/sorbose family IID component	2.45	5.21E-42
Cbei_0683	-	ABR32869.1	Radical SAM domain protein	2.40	2.85E-178
Cbei_4558	-	ABR36667.1	PTS system sorbose-specific transporter subunit IIC	2.34	1.01E-32
Cbei_1301	-	ABR33481.1	Membrane associated protein	2.30	7.00E-11
Cbei_0692	*aroH*	ABR32878.1	Phospho-2-dehydro-3-deoxyheptonate aldolase	2.24	1.05E-278
Cbei_0690	-	ABR32876.1	Radical SAM domain protein	2.12	1.77E-166
Cbei_0968	-	ABR33152.1	Polysaccharide biosynthesis protein	2.06	1.96E-12
Cbei_0688	-	ABR32874.1	Beta-lactamase domain-containing protein	2.03	6.72E-144
Cbei_3030	-	ABR35169.1	Radical SAM domain protein	2.01	4.74E-16
Cbei_2383	*xylA*	ABR34543.1	Xylose isomerase	2.00	1.24E-66

Cbei_4432	*mglC*	ABR36541.1	Monosaccharide-transporting ATPase	1.98	2.39E-19
Cbei_2534	*feoA*	ABR34690.1	FeoA family protein	1.95	6.46E-09
Cbei_4816	-	ABR36923.1	Hemerythrin-like metal-binding protein	1.92	2.85E-14
Cbei_3367	-	ABR35493.1	Sugar-specific permease, SgaT/UlaA	1.91	8.71E-05
Cbei_4433	*mglA*	ABR36542.1	ABC transporter	1.90	6.96E-80
Cbei_2385	*xylR*	ABR34545.1	ROK family protein	1.90	3.64E-68
Cbei_2122	-	ABR34289.1	Amino acid/peptide transporter	1.90	1.00E-120
Cbei_3876	-	ABR35990.1	PfkB domain protein/ ribokinase-like domain-containing protein	1.85	9.15E-12
Cbei_3123	*rbsA2*	ABR35259.1	ABC transporter	1.80	1.77E-57
Cbei_4931	*oppC*	ABR37037.1	Binding-protein-dependent transport systems inner membrane component	1.76	1.38E-09
Cbei_4007	*kdgT*	ABR36117.1	2-keto-3-deoxygluconate permease	1.75	5.11E-09
Cbei_2165	-	ABR34332.1	Hemerythrin-like metal-binding protein	1.69	3.57E-06
Cbei_4930	*oppD*	ABR37036.1	Peptide ABC transporter ATPase	1.68	3.38E-21
Cbei_0336	*srlA*	ABR32524.1	PTS system glucitol/sorbitol-specific transporter subunit IIC	1.67	8.70E-33
Cbei_2535	*feoB*	ABR34691.1	Ferrous iron transport protein B	1.66	2.47E-84
Cbei_2192	*gnd*	ABR34359.1	6-phosphogluconate dehydrogenase	1.65	0.004553097
Cbei_0028	*carA*	ABR32218.1	Carbamoyl phosphate synthase small subunit	1.60	4.45E-44
Cbei_0337	*srlE*	ABR32525.1	PTS system glucitol/sorbitol-specific transporter subunit IIBC	1.60	6.65E-48
Cbei_4833	-	ABR36940.1	Glycoside hydrolase	1.57	4.90E-39
Cbei_0450	-	ABR32638.1	Xylose isomerase domain protein TIM barrel	1.55	0.030993403
Cbei_4566	-	ABR36675.1	Beta-glucosidase	1.54	1.87E-14
Cbei_4681	-	ABR36789.1	L-fucose isomerase related protein	1.52	6.78E-59
Cbei_4567	-	ABR36676.1	ROK family protein	1.50	2.77E-16
Cbei_4149	*aspC*	ABR36259.1	Class I and II aminotransferase (aspartate aminotransferase)	1.49	1.47E-34
Cbei_3877	-	ABR35991.1	Sucrose-6-phosphate hydrolase	1.48	9.32E-05
Cbei_3539	-	ABR35661.1	Binding-protein-dependent transport systems inner membrane component	1.48	7.78E-35
Cbei_1752	*trpC*	ABR33924.1	Indole-3-glycerol-phosphate synthase	1.47	0.012413776
Cbei_1973	-	ABR34143.1	Selenate reductase subunit YgfK	1.47	3.90E-84
Cbei_0874	-	ABR33058.1	Hexapaptide repeat-containing transferase	1.47	5.09E-15
Cbei_1090	-	ABR33273.1	AMP-dependent synthetase and ligase	1.45	1.83E-69

Cbei_0029	*carB*	ABR32219.1	Carbamoyl phosphate synthase, large subunit	1.42	8.17E-80
Cbei_4932	*oppB*	ABR37038.1	Binding-protein-dependent transport systems inner membrane component	1.42	1.33E-20
Cbei_3325	-	ABR35452.1	ABC transporter	1.40	0.000572742
Cbei_0225	*dxs_3*	ABR32415.1	Transketolase, central region	1.38	2.07E-05
Cbei_0071	*nrdG*	ABR32261.1	Anaerobic ribonucleoside-triphosphate reductase activating protein	1.33	0.000689972
Cbei_4734	-	ABR36842.1	Galactoside O-acetyltransferase	1.32	0.002724399
Cbei_4674	-	ABR36782.1	Glycoside hydrolase family protein	1.31	1.90E-22
Cbei_2320	*treP*	ABR34480.1	PTS system trehalose-specific transporter subunit IIBC	1.28	1.86E-21
Cbei_1715	*pyrP*	ABR33887.1	Uracil-xanthine permease	1.26	2.97E-34
Cbei_4479	*glxK*	ABR36588.1	Glycerate kinase	1.26	9.21E-05
Cbei_1749	*trpE*	ABR33921.1	Anthranilate synthase component I	1.25	2.79E-06
Cbei_0262	-	ABR32451.1	ABC transporter	1.25	1.32E-94
Cbei_1009	*pfl*	ABR33193.1	Formate acetyltransferase	1.23	8.53E-37
Cbei_4685	-	ABR36793.1	PTS system lactose/cellobiose-specific transporter subunit IIA	1.21	0.005952426
Cbei_4540	-	ABR36649.1	Adenylate cyclase	1.21	0.032450444
Cbei_1111	*mltG*	ABR33293.1	Aminodeoxychorismate lyase	1.20	1.90E-66
Cbei_1974	-	ABR34144.1	Xanthine/uracil/vitamin C permease	1.20	3.97E-40
Cbei_2793	-	ABR34943.1	ABC transporter	1.20	7.26E-20
Cbei_1982	-	ABR34152.1	Aldehyde oxidase and xanthine dehydrogenase molybdopterin-binding subunit	1.20	7.22E-32
Cell wall/membrane/envelop biogenesis					
Cbei_3778	-	ABR35895.1	Glycosyl transferase family protein	5.19	0.001397647
Cbei_4556	-	ABR36665.1	Sugar isomerase (SIS)	2.67	2.73E-174
Cbei_4113	*alr*	ABR36223.1	Alanine racemase	2.60	8.77E-161
Cbei_1088	*algI*	ABR33271.1	Membrane bound O-acyl transferase, MBOAT family protein	1.78	3.24E-51
Cbei_4757	-	ABR36864.1	Undecaprenyl-phosphate galactose phosphotransferase	1.50	2.29E-29
Cbei_3197	-	ABR35327.1	Glycosyl transferase family protein	1.31	2.36E-79
Cbei_4758	-	ABR36865.1	Glycosyl transferase family protein	1.27	6.73E-24
Cbei_4277	*pseG*	ABR36387.1	Glycosyl transferase	1.27	1.52E-28
Cbei_0581	*ddl*	ABR32768.1	D-alanine--D-alanine ligase	1.20	4.40E-24
Cbei_4331	*dltB*	ABR36441.1	Membrane bound O-acyl transferase, MBOAT family protein	1.20	1.71E-38
Lipid biosynthesis & metabolism					
Cbei_4543	-	ABR36652.1	Coenzyme A transferase/propionate CoA-transferase	3.80	0.0000

Cbei_4544	*crt*	ABR36653.1	Enoyl-CoA hydratase/isomerase	3.71	2.23E-27
Cbei_4542	-	ABR36651.1	Acyl-CoA dehydrogenase domain protein	3.15	1.41E-210
Cbei_0684	-	ABR32870.1	AMP-dependent synthetase & ligase	2.40	6.10E-303
Cbei_0691	-	ABR32877.1	Acyl-ACP thioesterase	2.26	6.05E-166
Cbei_0687	-	ABR32873.1	4'-phosphopantetheinyl transferase	2.24	3.81E-184
Cbei_4032	-	ABR36142.1	Beta-ketoacyl-acyl-carrier-protein synthase I	2.22	4.12E-49
Cbei_0681	-	ABR32867.1	Thioesterase	2.18	1.48E-180
Cbei_0686	-	ABR32872.1	AMP-dependent synthetase and ligase	2.17	0.0000
Cbei_0340	-	ABR32528.1	Short-chain dehydrogenase/reductase (SDR)	1.95	5.71E-07
Cbei_1305	-	ABR33485.1	Lysophospholipase	1.73	6.01E-22
Cbei_1072	*fabF*	ABR33256.1	Beta-ketoacyl synthase-like protein	1.34	3.41E-73
Cbei_1074	*fabZ*	ABR33258.1	Beta-hydroxyacyl-(acyl-carrier-protein) dehydratase FabZ	1.22	1.06E-52
Cbei_1071	*fabG*	ABR33255.1	3-oxoacyl-(acyl-carrier-protein) reductase	1.20	3.75E-31
Cbei_0248	-	ABR32438.1	Oleoyl-(acyl-carrier-protein) hydrolase	1.20	2.04E-05
Cell cycle control and mitosis					
Cbei_0422	*spoIID*	ABR32610.1	Sporulation stage II protein D	2.16	3.91E-44
Cbei_4763	-	ABR36870.1	Cell wall binding repeat-containing protein	1.74	2.46E-77
Cbei_3970	*sspH*	ABR36080.1	Small acid-soluble spore H family protein	1.68	0.000248557
Cbei_2337	-	ABR34497.1	Cell wall binding repeat-containing protein	1.67	0.000248557
Cbei_2337	-	ABR34497.1	Cell wall binding repeat-containing protein	1.66	0.012025806
Cbei_1692	*spoIIIAA*	ABR33864.1	Sporulation stage III, protein AA	1.40	7.19E-07
Cbei_0919	-	ABR33103.1	Cell wall binding repeat-containing protein	1.38	1.11E-14
Cbei_4719	-	ABR36827.1	Cell wall binding repeat-containing protein	1.36	1.65E-102
Cbei_4766	-	ABR36873.1	Cell wall binding repeat-containing protein	1.20	3.71E-41
Cbei_0560	-	ABR32747.1	ExsB family protein	1.20	5.53E-11
Stress response					
Cbei_4586	-	ABR36694.1	Lantibiotic modifying-like protein	2.91	5.69E-219
Cbei_0260		ABR32449.1	EmrB/QacA family drug resistance transporter	1.20	2.71E-77
Unknown functions					
Cbei_3040	-	ABR35179.1	Hypothetical protein	5.30	0.000901115
Cbei_2267	-	ABR34428.1	Hypothetical protein	4.45	0.017980963
Cbei_5021	-	ABR37127.1	Hypothetical protein	4.24	0.031477913
Cbei_1303	-	ABR33483.1	Hypothetical protein	3.57	1.18E-199
Cbei_3555	-	ABR35677.1	Hypothetical protein	3.30	1.15E-21

Cbei_4587	-	ABR36695.1	Hypothetical protein	3.16	2.85E-172
Cbei_2300	-	ABR34460.1	Hypothetical protein	2.87	0.030325013
Cbei_3554	-	ABR35676.1	Conserved hypothetical protein	2.74	2.46E-22
Cbei_0030	-	ABR32220.1	Hypothetical protein	2.72	1.31E-06
Cbei_4074	-	ABR36184.1	Hypothetical protein	2.71	3.78E-82
Cbei_3755	-	ABR35872.1	Hypothetical protein	2.58	3.40E-06
Cbei_4675	-	ABR36783.1	Hypothetical protein	2.57	2.81E-33
Cbei_0682	-	ABR32868.1	Hypothetical protein	2.46	1.93E-54
Cbei_4637	-	ABR36745.1	Conserved hypothetical protein	2.40	3.82E-53
Cbei_0689	-	ABR32875.1	Hypothetical protein	2.34	6.20E-108
Cbei_0908	-	ABR33092.1	HK97 family phage prohead protease	2.28	4.99E-09
Cbei_2167	-	ABR34334.1	Hypothetical protein	2.27	0.001017436
Cbei_4583	-	ABR36691.1	Hypothetical protein	2.27	1.49E-67
Cbei_2168	-	ABR34335.1	Hypothetical protein	2.11	0.000554935
Cbei_2166	-	ABR34333.1	Hypothetical protein	2.11	5.21E-15
Cbei_3556	-	ABR35678.1	Hypothetical protein	2.07	1.75E-31
Cbei_5016	-	ABR37122.1	Hypothetical protein	2.00	4.05E-13
Cbei_0911	-	ABR33095.1	Phage head-tail adaptor	1.98	0.001167312
Cbei_2169	-	ABR34336.1	Hypothetical protein	1.92	3.34E-07
Cbei_3687	-	ABR35805.1	Hypothetical protein	1.90	3.02E-97
Cbei_4033	-	ABR36143.1	Hypothetical protein	1.83	2.35E-08
Cbei_0303	-	ABR32491.1	Conserved hypothetical protein	1.78	6.46E-06
Cbei_0926	-	ABR33110.1	Hypothetical protein	1.74	6.28E-45
Cbei_0914	-	ABR33098.1	Hypothetical protein	1.72	1.66E-19
Cbei_0928	-	ABR33112.1	Hypothetical protein	1.72	0.000323791
Cbei_3470	-	ABR35595.1	Hypothetical protein	1.70	4.60E-13
Cbei_2170	-	ABR34337.1	Hypothetical protein	1.70	2.76E-06
Cbei_0906	-	ABR33090.1	Phage terminase	1.70	1.34E-24
Cbei_3688	-	ABR35806.1	Hypothetical protein	1.68	3.36E-86
Cbei_0924	-	ABR33108.1	Hypothetical protein	1.63	6.09E-43
Cbei_0912	-	ABR33096.1	Hypothetical protein	1.63	2.80E-10
Cbei_5014	-	ABR37120.1	Hypothetical protein	1.60	1.24E-11
Cbei_4248	-	ABR36358.1	Hypothetical protein	1.57	4.37E-09
Cbei_0249	-	ABR32439.1	Hypothetical protein	1.56	1.62E-15
Cbei_3557	-	ABR35679.1	TROVE domain protein	1.55	1.28E-17
Cbei_4812	-	ABR36919.1	Hypothetical protein	1.54	0.006022637
Cbei_3465	-	ABR35590.1	Hypothetical protein	1.49	1.63E-12
Cbei_1089	-	ABR33272.1	Hypothetical protein	1.48	5.41E-46
Cbei_0913	-	ABR33097.1	Hypothetical protein	1.47	3.35E-05
Cbei_4212	-	ABR36322.1	Hypothetical protein	1.47	0.00213873
Cbei_4469	-	ABR36578.1	SCP-like extracellular	1.46	0.000265992
Cbei_4364	-	ABR36474.1	Hypothetical protein	1.46	2.70E-15
Cbei_0280	-	ABR32468.1	Hypothetical protein	1.44	1.79E-111
Cbei_5038	*ycfA*	ABR37144.1	Hypothetical protein	1.44	2.63E-10
Cbei_3686	-	ABR35804.1	Hypothetical protein	1.43	2.83E-67
Cbei_0917	-	ABR33101.1	Hypothetical protein	1.42	5.26E-08
Cbei_2062	-	ABR34230.1	Hypothetical protein	1.41	0.00961394
Cbei_0892	-	ABR33076.1	Hypothetical protein	1.40	1.96E-13
Cbei_5022	-	ABR37128.1	Hypothetical protein	1.40	1.09E-114
Cbei_0995	-	ABR33179.1	Tetratricopeptide TPR_2 repeat protein	1.40	5.06E-38
Cbei_0903	-	ABR33087.1	Prophage LambdaBa04, GP54	1.39	0.031486639

Cbei_0905	-	ABR33089.1	Hypothetical protein	1.36	7.07E-05
Cbei_0927	-	ABR33111.1	Hypothetical protein	1.36	0.002877667
Cbei_0893	-	ABR33077.1	Hypothetical protein	1.33	1.19E-10
Cbei_4075	-	ABR36185.1	Hypothetical protein	1.31	7.58E-05
Cbei_0925	-	ABR33109.1	Phage-like element pbsx protein XkdT	1.30	1.12E-07
Cbei_1952	-	ABR34122.1	Hypothetical protein	1.28	0.001504484
Cbei_0757	-	ABR32941.1	Hypothetical protein	1.28	1.21E-10
Cbei_4114	-	ABR36224.1	HSR1-like GTP-binding protein	1.27	1.28E-70
Cbei_4628	-	ABR36736.1	Hypothetical protein	1.26	6.44E-66
Cbei_3685	-	ABR35803.1	Hypothetical protein	1.26	1.56E-33
Cbei_0918	-	ABR33102.1	Hypothetical protein	1.25	8.05E-13
Cbei_0910	-	ABR33094.1	Hypothetical protein	1.24	0.011347769
Cbei_0263	-	ABR32452.1	Hypothetical protein	1.21	1.45E-72
Cbei_0923	-	ABR33107.1	Baseplate J family protein	1.20	6.33E-12
Cbei_2749	-	ABR34900.1	Conserved hypothetical protein	1.20	0.000120714
Cbei_0208	-	ABR32398.1	Hypothetical protein	1.20	1.09E-07
Cbei_0920	-	ABR33104.1	Hypothetical protein	1.20	3.09E-12
