# Supplementary material for: Transcriptomic characterization of recombinant Clostridium beijerinckii NCIMB 8052 expressing methylglyoxal synthase and glyoxal reductase from Clostridium pasteurianum ATCC 6013
Source: Appl Environ Microbiol. 2024 Sep 11;90(10):e01012-24. doi: 10.1128/aem.01012-24 (PMC11497831; doi:10.1128/aem.01012-24)
Supplement: Table S2 — Complete list of downregulated genes. [file aem.01012-24-s0002.pdf]

Table S2 Genes downregulated in *C. beijerinckii*_mgsA + mgR relative to *C. beijerinckii*_p459.

Gene ID	Gene symbol	Protein ID	Protein function	Fold change (Log_2)	*p* value
**Coenzyme metabolism**					
Cbei_0792	*nadA*	ABR32976.1	Quinolinate synthetase	-7.14	9.1E-237
Cbei_0794	*nadC*	ABR32978.1	Nicotinate-nucleotide pyrophosphorylase/quinolinate phosphoribosyl transferase	-5.90	0.0000
Cbei_0793	*nadB*	ABR32977.1	L-aspartate oxidase	-5.50	8.71E-112
Cbei_5039	*pdxT*	ABR37145.1	Glutamine amidotransferase	-3.72	5.31E-146
Cbei_5040	*pdxS*	ABR37146.1	Pyridoxal biosynthesis lyase	-3.60	1.07E-67
Cbei_1510	*ilvD*	ABR33686.1	Dihydroxy-acid dehydratase	-3.03	5.04E-272
Cbei_3792	*moaC*	ABR35909.1	Molybdenum cofactor biosynthesis protein C	-1.97	8.56E-06
**Energy production and conservation**					
Cbei_0795	-	ABR32979.1	4Fe-4S ferredoxin iron-sulfur binding domain protein	-5.21	0.00
Cbei_0620	*nifD1*	ABR32807.1	Nitrogenase/oxidoreductase	-4.42	2.82E-192
Cbei_0621	-	ABR32808.1	Nitrogenase/oxidoreductase	-4.10	0.0000
Cbei_3759	-	ABR35876.1	FAD-linked oxidase domain protein	-3.80	0.0000
Cbei_2182	*pyrK_1*	ABR34349.1	Ferredoxin-$NADP^{(+)}$ reductase subunit alpha	-3.30	1.79E-146
Cbei_3496	-	ABR35619.1	FAD linked oxidase domain protein	-3.30	0.0000
Cbei_0628	-	ABR32815.1	Radical SAM domain-containing protein	-3.17	4.92E-62
Cbei_2183	*gltA*	ABR34350.1	Oxidoreductase/glutamate synthase	-3.10	0.0000
Cbei_0631	-	ABR32818.1	Nitrogenase	-2.82	1.43E-56
Cbei_0627	-	ABR32814.1	Dinitrogenase iron-molybdenum cofactor biosynthesis protein	-2.80	5.63E-06
Cbei_2181	*adh*	ABR34348.1	Iron-containing alcohol dehydrogenase (butanol dehydrogenase)	-2.74	1.72E-285
Cbei_3643	-	ABR35762.1	Nitroreductase	-2.01	1.30E-92
Cbei_2518	-	ABR34674.1	Aldehyde dehydrogenase	-2.00	8.39E-158
Cbei_1012	*rubR2*	ABR33196.1	Rubredoxin-type Fe(Cys)4 protein	-1.77	1.15E-60
Cbei_3544	-	ABR35666.1	4Fe-4S ferredoxin	-1.75	0.00621056
Cbei_3046	-	ABR35185.1	NADPH-dependent FMN reductase	-1.75	2.63E-08
Cbei_0632	-	ABR32819.1	Nitrogenase	-1.60	2.99E-13
Cbei_1999	*nifH*	ABR34169.1	Nitrogenase iron protein	-1.54	0.000340251
Cbei_3328	-	ABR35455.1	FAD-dependent pyridine nucleotide-disulphide oxidoreductase	-1.46	3.25E-127
Cbei_2243	-	ABR34407.1	Alcohol dehydrogenase, zinc-binding domain protein; GroES domain protein	-1.45	1.83E-16
Cbei_3895	-	ABR36009.1	NADPH-dependent FMN reductase	-1.40	0.004049085
Cbei_2002	*nifD*	ABR34172.1	Nitrogenase molybdenum-iron protein alpha chain	-1.37	8.94E-75
Cbei_0037	*pflX*	ABR32227.1	Radical SAM domain-containing protein	-1.26	2.03E-40
Cbei_2649	-	ABR34803.1	Ferric reductase domain-containing protein	-1.23	8.63E-15

Cbei_3642	*korB*	ABR35761.1	2-oxoglutarate ferredoxin oxidoreductase subunit beta	-1.22	8.38E-12
Cbei_3215	-	ABR35345.1	Nitroreductase	-1.20	2.94E-05
Cbei_1852	*iscU*	ABR34023.1	NifU family SUF system FeS assembly protein	-1.20	8.90E-37
Cbei_4835	-	ABR36942.1	4Fe-4S ferredoxin	-1.20	7.17E-15
**Intracellular trafficking and secretion**					
Cbei_3739	-	ABR35856.1	Metallophosphoesterase	-1.90	2.35E-56
Cbei_4344	-	ABR36454.1	Sodium:dicarboxylate symporter	-1.70	3.61E-105
**Secondary structure**					
Cbei_2603	-	ABR34759.1	Collagen triple helix repeat	-4.60	0.00
Cbei_2587	-	ABR34743.1	Collagen triple helix repeat	-4.40	0.00
Cbei_2588	-	ABR34744.1	Collagen triple helix repeat	-3.50	0.00
Cbei_2980	-	ABR35120.1	Multicopper oxidase, type 3	-3.15	4.81E-236
Cbei_2852	*phzA*	ABR34999.1	Isochorismatase hydrolase	-1.61	9.96E-05
**Transcription, replication and repair**					
Cbei_3456	-	ABR35581.1	XRE family transcriptional regulator	-3.90	0.000111359
Cbei_4069	-	ABR36179.1	Iron-dependent transcription repressor	-3.20	7.54E-151
Cbei_4997	-	ABR37103.1	XRE family transcriptional regulator	-3.20	8.86E-10
Cbei_3743	-	ABR35860.1	RNA polymerase, sigma-24 subunit, ECF subfamily	-2.80	6.67E-139
[3]Cbei_2219	-	ABR34385.1	AbrB family transcriptional regulator	-2.60	0.000436535
Cbei_1424	-	ABR33602.1	Serine/threonine kinase related protein	-2.53	1.11E-70
Cbei_1678	-	ABR33851.1	XRE family transcriptional regulator	-2.32	0.000171307
Cbei_3479	-	ABR35603.1	XRE family transcriptional regulator	-2.25	8.43E-10
Cbei_3198	-	ABR35328.1	Prophage antirepressor	-2.10	9.15E-06
Cbei_3533	-	ABR35655.1	HxlR family transcriptional regulator	-1.91	9.71E-51
Cbei_1826	*sigI*	ABR33997.1	RNA polymerase sigma factor SigI	-1.90	1.71E-240
Cbei_1598	-	ABR33772.1	RpiR family transcriptional regulator	-1.90	1.52E-24
Cbei_3301	-	ABR35428.1	MarR family transcriptional regulator	-1.85	2.36E-42
Cbei_3047	-	ABR35186.1	Xylan 1,4-β-xylosidase (helix-turn-helix- domain containing protein, AraC type; glycoside hydrolase, family 39)	-1.80	5.54E-56
Cbei_3732		ABR35849.1	AAA ATPase	-1.74	3.81E-129
Cbei_2303	-	ABR34463.1	ECF subfamily RNA polymerase sigma-24 factor	-1.66	4.44E-05
Cbei_1623	-	ABR33797.1	Primosome subunit DnaD	-1.60	5.24E-12
Cbei_1635	-	ABR33809.1	Phage-related terminase small subunit-like protein	-1.57	7.46E-14
Cbei_3631	-	ABR35751.1	LysR family transcriptional regulator	-1.55	4.88E-30
Cbei_2951	-	ABR35092.1	Two component transcriptional regulator	-1.53	3.40E-07
Cbei_3937	-	ABR36047.1	GCN5-related N-acetyltransferase	-1.53	0.002376342
Cbei_3644	-	ABR35763.1	TetR family transcriptional regulator	-1.53	6.83E-22
Cbei_3735	-	ABR35852.1	XRE family transcriptional regulator	-1.50	1.41E-06
Cbei_1373	-	ABR33553.1	Phage integrase family protein	-1.50	2.69E-23
Cbei_1078	*hutP*	ABR33262.1	HutP protein	-1.50	2.67E-07
Cbei_1115	*sigK*	ABR33297.1	RNA polymerase (sporulation sigma factor SigK)	-1.48	1.16E-91
Cbei_4998	-	ABR37104.1	ATPase-like protein	-1.45	5.45E-17

Cbei_3513	-	ABR35636.1	ArsR family transcriptional regulator	-1.44	0.003973525
Cbei_3706	-	ABR35823.1	LuxR family transcriptional regulator	-1.44	9.52E-09
Cbei_3616	-	ABR35736.1	XRE family transcriptional regulator	-1.43	1.22E-11
Cbei_3108	*ydgH*	ABR35245.1	MMPL domain protein	-1.40	3.08E-34
Cbei_2924	-	ABR35067.1	AcrR family transcriptional regulator	-1.40	0.031313736
Cbei_1132	*phoU*	ABR33314.1	Phosphate uptake regulator PhoU	-1.40	1.73E-13
Cbei_2721		ABR34872.1	MerR family transcriptional regulator	-1.35	0.002758724
Cbei_5082	*yybT*	ABR37188.1	Phosphoesterase domain-containing protein	-1.33	7.58E-151
Cbei_3483	-	ABR35607.1	XRE family transcriptional regulator	-1.32	6.41E-06
Cbei_2221	-	ABR34386.1	BadM/Rrf2 family transcriptional regulator	-1.32	0.000365992
Cbei_2557	*xerC*	ABR34713.1	Phage integrase family protein	-1.31	1.88E-35
Cbei_2866	-	ABR35013.1	TetR family transcriptional regulator	-1.30	7.04E-24
Cbei_5031	*levR3*	ABR37137.1	Sigma-54 factor interaction domain-containing protein	-1.28	4.14E-64
Cbei_1253	-	ABR33434.1	TetR family transcriptional regulator	-1.27	1.98E-35
Cbei_3291	-	ABR35418.1	GCN5-related N-acetyltransferase	-1.24	0.000873132
Cbei_0120	*ctsR*	ABR32310.1	Transcriptional repressor CtsR	-1.21	2.31E-11
Cbei_0886	-	ABR33070.1	XRE family transcriptional regulator	-1.20	1.21E-05
**Translation**					
Cbei_1881	-	ABR34052.1	Glucose-inhibited division protein A	-2.71	1.22E-232
Cbei_2108	*arsR*	ABR34275.1	Regulatory protein, ArsR	-2.70	2.24E-15
Cbei_1933	*gidA*	ABR34103.1	Glucose-inhibited division protein A	-1.80	7.45E-62
Cbei_2127	*gatC*	ABR34294.1	Glutamyl-tRNA(Gln) amidotransferase subunit C	-1.44	4.04E-13
Cbei_3844	-	ABR35959.1	GCN5-related N-acetyltransferase	-1.42	0.003692953
Cbei_0873	-	ABR33057.1	Metallophosphoesterase	-1.22	3.91E-05
**Post-translational modification/protein turnover/chaperone functions**					
Cbei_4418	-	ABR36527.1	RNA-binding region-containing protein (RNP-1)	-3.30	3.09E-281
Cbei_4123	*hsp*	ABR36233.1	Heat shock protein Hsp20	-2.72	0.0000
Cbei_1254	*lon*	ABR33435.1	ATP-dependent protease La	-2.64	0.0000
Cbei_0645	*clpB*	ABR32832.1	ATPase AAA-2 domain protein	-1.81	1.14E-256
Cbei_0328	*groS*	ABR32516.1	Chaperonin Cpn10	-1.63	0.018223099
Cbei_0329	*groL*	ABR32517.1	Chaperonin GroEL	-1.30	5.20E-108
**Cell motility and signal transduction**					
Cbei_3953	-	ABR36063.1	Methyl-accepting chemotaxis sensory transducer	-2.90	1.21E-66
Cbei_1652	-	ABR33826.1	SH3 type 3 domain-containing protein	-2.13	3.62E-15
Cbei_3954	-	ABR36064.1	CheW protein	-2.10	5.35E-05
Cbei_2850	-	ABR34997.1	Methyl-accepting chemotaxis protein	-2.10	2.39E-08
Cbei_3386	-	ABR35512.1	Periplasmic ligand-binding sensor protein	-1.84	6.38E-187
Cbei_2950	-	ABR35091.1	Integral membrane sensor signal transduction histidine kinase	-1.84	6.79E-42
Cbei_3303	-	ABR35430.1	Diguanylate cyclase/phosphodiesterase	-1.63	1.99E-38
Cbei_4800	*artP*	ABR36907.1	Extracellular solute-binding protein, family 3	-1.60	1.75E-27
Cbei_2111	*arsC*	ABR34278.1	Protein tyrosine phosphatase	-1.54	0.000464983

Cbei_3050	-	ABR35188.1	Response regulator receiver protein	-1.53	3.76E-17
Cbei_2849	-	ABR34996.1	Methyl-accepting chemotaxis sensory transducer	-1.46	2.16E-30
Cbei_2912	-	ABR35055.1	Methyl-accepting chemotaxis sensory transducer	-1.33	1.18E-06
Cbei_3314	-	ABR35441.1	Diguanylate cyclase	-1.30	9.71E-29
Cbei_1723	-	ABR33895.1	Methyl-accepting chemotaxis sensory transducer	-1.20	1.76E-10
Cbei_2919	-	ABR35062.1	Two component transcriptional regulator	-1.20	5.09E-07
**Nutrient/nucleotide transport and metabolism**					
Cbei_0622	-	ABR32809.1	Cysteine synthase	-3.90	2.70E-92
Cbei_0626	-	ABR32813.1	NLPA lipoprotein	-3.33	6.03E-74
Cbei_0625	-	ABR32812.1	Binding-protein-dependent transport system inner membrane protein	-3.23	4.46E-66
Cbei_1510	*ilvD*	ABR33686.1	Dihydroxy-acid dehydratase	-3.00	5.04E-272
Cbei_0629	*metC*	ABR32816.1	Cystathionine gamma-synthase	-3.0	5.47E-81
Cbei_2954	-	ABR35095.1	Cupin 2, conserved barrel domain protein	-2.83	1.73E-239
Cbei_2069	*cotJC*	ABR34237.1	Manganese containing catalase (includes spore coat peptide assembly protein CotJC)	-2.80	1.97E-192
Cbei_0933	-	ABR33117.1	ABC transporter substrate-binding protein	-2.80	2.48E-214
Cbei_3709	*cbiN*	ABR35826.1	Cobalt/cobalamin transport protein CbiN	-2.73	1.10E-13
Cbei_3279	-	ABR35408.1	Cation diffusion facilitator family transporter	-2.64	3.61E-105
Cbei_2597	-	ABR34753.1	DegT/DnrJ/EryC1/StrS aminotransferase	-2.64	6.34E-143
Cbei_2419	*Ppk1*	ABR34577.1	Polyphosphate kinase 1	-2.61	4.25E-139
Cbei_0585	*csd*	ABR32772.1	Cysteine desulfurase	-2.60	1.20E-217
Cbei_4470	-	ABR36579.1	Phosphotransferase domain-containing protein	-2.60	6.39E-117
Cbei_3257	-	ABR35386.1	[#]Rubrerythrin	-2.60	2.70E-90
Cbei_0318	*manC*	ABR32506.1	Mannose-1-phosphate guanylyltransferase (GDP)	-2.60	1.87E-176
Cbei_0934	*ssuB_2*	ABR33118.1	ABC transporter	-2.54	4.57E-96
Cbei_4067	-	ABR36177.1	Xanthine/uracil/vitamin C permease	-2.53	6.40E-186
Cbei_0935	*ssuC_2*	ABR33119.1	Binding-protein-dependent transport system inner membrane protein	-2.53	5.98E-213
Cbei_3267	-	ABR35396.1	Glycoside hydrolase	-2.50	6.66E-50
Cbei_3049	-	ABR35187.1	ABC transporter	-2.40	1.28E-171
[3]Cbei_1131	*phoU*	ABR33313.1	Phosphate uptake regulator PhoU	-2.40	5.77E-64
Cbei_3300	-	ABR35427.1	Multidrug ABC transporter ATPase	-2.40	2.85E-117
Cbei_0760	-	ABR32944.1	Membrane spanning protein	-2.40	8.23E-75
Cbei_0591	-	ABR32778.1	Glycoside hydrolase	-2.40	4.28E-229
Cbei_0707	-	ABR32891.1	Major facilitator superfamily transporter	-2.34	2.99E-37
Cbei_1034	*asnB*	ABR33218.1	Asparagine synthase	-2.33	1.08E-203
Cbei_1759	*gpmA*	ABR33931.1	Phosphoglycerate mutase	-2.31	1.78E-13
Cbei_2590	-	ABR34746.1	Capsular polysaccharide biosynthesis-like protein	-2.30	3.46E-194
Cbei_0759	-	ABR32943.1	Serine--pyruvate transaminase	-2.30	2.81E-92

Cbei_3543	-	ABR35665.1	O-acetylhomoserine aminocarboxypropyltransferase	-2.25	6.39E-28
Cbei_1130	*pstB*	ABR33312.1	Phosphate ABC transporter ATPase	-2.21	1.48E-70
Cbei_0630	*mccA*	ABR32817.1	Pyridoxal-5'-phosphate-dependent protein subunit beta (cysteine synthase)	-2.20	1.61E-40
Cbei_3299	-	ABR35426.1	Multidrug ABC transporter ATPase	-2.20	4.49E-199
Cbei_1127	*pstS*	ABR33309.1	Phosphate binding protein	-2.20	3.27E-189
Cbei_1129	*pstA*	ABR33311.1	Phosphate ABC transporter permease	-2.20	6.20E-123
Cbei_4704	*malH*	ABR36812.1	Glycoside hydrolase	-2.20	1.86E-88
Cbei_3277	-	ABR35406.1	Class II aldolase/adducin family protein	-2.11	3.22E-190
Cbei_0623	*nifH*	ABR32810.1	Nitrogenase iron protein	-2.10	5.15E-11
Cbei_0624	*metN1*	ABR32811.1	ABC transporter	-2.10	2.76E-08
Cbei_1054	*purE*	ABR33238.1	Phosphoribosylaminoimidazole carboxylase catalytic subunit	-2.10	5.02E-59
Cbei_2870	*opuCC*	ABR35016.1	Glycine/betaine ABC transporter substrate-binding protein	-2.05	2.67E-120
Cbei_0877	-	ABR33061.1	ABC transporter substrate-binding protein	-2.05	1.71E-28
Cbei_0464	*ribU*	ABR32652.1	Putative membrane transport protein	-2.05	4.49E-129
Cbei_2459	*kdgT*	ABR34617.1	2-keto-3-deoxygluconate permease	-2.02	8.86E-40
Cbei_0859	*ntpR*	ABR33043.1	Peptidase C26	-2.02	2.24E-145
Cbei_3332	*tauA*	ABR35459.1	Aliphatic sulfonate ABC transporter periplasmic	-2.02	4.40E-181
Cbei_1128	*pstC*	ABR33310.1	Phosphate ABC transporter permease	-2.00	9.86E-127
Cbei_4707	-	ABR36815.1	Glycosyl transferase family protein	-2.00	8.48E-96
Cbei_1992	*modA*	ABR34162.1	Molybdenum ABC transporter periplasmic molybdate-binding protein	-1.97	9.04E-28
Cbei_3353	-	ABR35479.1	Glycoside hydrolase	-1.96	2.45E-18
Cbei_2871	*proV*	ABR35017.1	Glycine/betaine ABC transporter ATPase	-1.93	1.25E-73
Cbei_2907	-	ABR35052.1	PTS system N-acetylgalactosamine-specific transporter subunit IIC	-1.91	0.015775789
Cbei_0610	-	ABR32797.1	ABC transporter	-1.90	1.33E-07
Cbei_2112	*arsB*	ABR34279.1	Arsenical-resistance protein	-1.90	7.81E-81
Cbei_0609	*metY*	ABR32796.1	O-acetylhomoserine/O-acetylserine sulfhydrylase	-1.83	4.16E-14
Cbei_0462	*eda*	ABR32650.1	2-dehydro-3-deoxyphosphogluconate aldolase/4-hydroxy-2-oxoglutarate aldolase	-1.80	2.14E-73
Cbei_4070	*mntH*	ABR36180.1	Manganese transport protein MntH	-1.80	4.31E-60
Cbei_1440	-	ABR33618.1	Extracellular solute-binding protein	-1.80	1.90E-113
Cbei_0380	-	ABR32568.1	PTS system lactose/cellobiose-specific transporter subunit IIA	-1.80	6.21E-11
Cbei_3979	*appC*	ABR36089.1	Binding-protein-dependent transport system inner membrane protein	-1.71	2.62E-20
Cbei_3331	-	ABR35458.1	ABC transporter	-1.70	1.26E-168
Cbei_1795	*dapB*	ABR33966.1	Dihydrodipicolinate reductase	-1.66	3.18E-88
Cbei_0978	-	ABR33162.1	Major facilitator superfamily MFS_1	-1.65	3.81E-28

Cbei_2957	-	ABR35098.1	Cation diffusion facilitator family transporter	-1.61	1.42E-30
Cbei_0457	-	ABR32645.1	DeoR family transcriptional regulator	-1.60	1.01E-06
Cbei_3333	-	ABR35460.1	Binding-protein-dependent transport systems inner membrane component	-1.60	3.41E-76
Cbei_3980	-	ABR36090.1	Binding-protein-dependent transport systems inner membrane component	-1.56	7.42E-29
Cbei_4705	*glvC*		PTS system alpha-glucoside-specific transporter subunit IIBC	-1.53	6.76E-32
Cbei_3370	-	ABR35496.1	Ribose/galactose isomerase	-1.52	2.04E-10
Cbei_2174	*spxA*	ABR34341.1	Arsenate reductase-like protein	-1.50	1.46E-10
Cbei_3981	-	ABR36091.1	Extracellular solute-binding protein	-1.48	1.48E-52
Cbei_2769	-	ABR34920.1	Extracellular ligand-binding receptor	-1.47	0.001840255
Cbei_4798	-	ABR36905.1	Ribokinase-like domain-containing protein (PfkB domain protein)	-1.46	2.14E-15
Cbei_2468	*glgX*	ABR34626.1	Glycogen debranching enzyme GlgX	-1.45	3.85E-50
Cbei_2599	*csd2*	ABR34755.1	Cysteine desulfurase family protein	-1.40	1.10E-70
Cbei_0381	-	ABR32569.1	ABC-type sugar transport system periplasmic component-like protein	-1.38	2.68E-44
Cbei_2458	*kduI*	ABR34616.1	4-deoxy-L-threo-5-hexosulose-uronate ketol-isomerase	-1.38	1.48E-17
Cbei_2598	-	ABR34754.1	Amine oxidase	-1.33	2.59E-82
Cbei_2781	-	ABR34932.1	MATE efflux family protein	-1.33	2.07E-14
Cbei_0518	*asd*	ABR32706.1	Aspartate-semialdehyde dehydrogenase	-1.33	5.33E-33
Cbei_4787	*lysA*	ABR36894.1	Diaminopimelate decarboxylase	-1.33	1.51E-34
Cbei_1666	-	ABR33840.1	Glycoside hydrolase	-1.33	1.33E-06
Cbei_3728	*lyc*	ABR35845.1	Glycoside hydrolase	-1.31	2.84E-10
Cbei_1796	*dapA*	ABR33967.1	Dihydrodipicolinate synthase	-1.30	2.78E-94
Cbei_4339	-	ABR36449.1	Nucleotidyl transferase	-1.28	3.72E-58
Cbei_4338	*aepX*	ABR36448.1	Cytidyltransferase-related domain	-1.27	1.16E-79
Cbei_4194	*sbp*	ABR36304.1	Sulfate ABC transporter substrate-binding protein	-1.27	9.16E-08
Cbei_1053	*purL*	ABR33237.1	Phosphoribosylformylglycinamidine synthase	-1.27	2.23E-79
Cbei_0231	-	ABR32421.1	Binding-protein-dependent transport system inner membrane protein	-1.24	1.20E-71
Cbei_3561	*purH*	ABR35683.1	5-aminoimidazole-4-carboxamide ribonucleotide transformylase	-1.22	2.56E-08
Cbei_2853	-	ABR35000.1	Major facilitator superfamily MFS_1	-1.22	9.51E-09
Cbei_5034	*amt*	ABR37140.1	Ammonium transporter	-1.20	2.34E-16
Cbei_4512	-	ABR36621.1	Phosphoribulokinase/uridine kinase	-1.20	2.04E-33
Cbei_0306	-	ABR32494.1	ATPase P	-1.20	3.20E-61
Cbei_4670	-	ABR36778.1	6-phospho-beta-glucosidase	-1.20	0.000100267
Cbei_0461	-	ABR32649.1	PfkB domain protein	-1.20	8.30E-15
Cbei_2426	*pbuX*	ABR34584.1	Uracil-xanthine permease	-1.20	1.77E-31
Cbei_3704	*fhuC*	ABR35821.1	ABC transporter	-1.20	2.82E-13
Cbei_0232	-	ABR32422.1	Binding-protein-dependent transport systems inner membrane component	-1.20	1.96E-52
Cbei_4669	-	ABR36777.1	Glycoside hydrolase	-1.20	2.75E-06
**Cell wall/membrane/envelop biogenesis**					
Cbei_0386	-	ABR32574.1	Glycosyl transferase, group 1	-3.00	0.0000
Cbei_2589	*sunS*	ABR34745.1	Glycosyl transferase, family 2	-2.94	0.0000

Cbei_4708	*wbpA1*	ABR36816.1	UDP-glucose 6-dehydrogenase	-2.90	4.62E-233
Cbei_1437	-	ABR33615.1	Lipoprotein	-2.61	4.90E-94
Cbei_0318	*manC*	ABR32506.1	Mannose-1-phosphate guanylyltransferase (GDP)	-2.60	1.87E-176
[++]Cbei_2602	*rfbA*	ABR34758.1	Glucose-1-phosphate thymidylyltransferase	-2.50	1.43E-184
Cbei_2596	-	ABR34752.1	WxcM domain-containing protein	-2.50	1.07E-77
Cbei_2585	-	ABR34741.1	Group 1 glycosyl transferase	-2.43	4.27E-126
Cbei_0036	*sleC*	ABR32226.1	Peptidoglycan binding domain-containing protein	-2.40	2.29E-280
Cbei_2586	-	ABR34742.1	Glycosyl transferase	-2.40	2.51E-189
Cbei_0382	-	ABR32570.1	Group 1 glycosyl transferase	-2.30	7.94E-180
Cbei_2601	*rfbC*	ABR34757.1	dTDP-4-dehydrorhamnose 3,5-epimerase	-2.11	1.41E-90
Cbei_3673	-	ABR35791.1	Penicillin amidase	-1.96	1.82E-87
Cbei_2604	-	ABR34760.1	glycosyl transferase, family 2	-1.76	2.56E-105
●Cbei_1242	-	ABR33424.1	NLP/P60 protein	-1.75	2.71E-169
Cbei_2579	*rfbD*	ABR34735.1	dTDP-4-dehydrorhamnose reductase	-1.72	2.83E-80
Cbei_4799	-	ABR36906.1	Sugar isomerase (SIS)	-1.66	8.76E-18
Cbei_3936	-	ABR36046.1	Cell wall binding repeat-containing protein	-1.63	3.42E-65
Cbei_2578	*rfbB*	ABR34734.1	dTDP-glucose 4,6-dehydratase	-1.48	2.93E-66
Cbei_2970		ABR35111.1	Prolipoprotein diacylglyceryl transferase	-1.34	0.001355588
Cbei_0421	*murA*	ABR32609.1	UDP-N-acetylglucosamine 1-carboxyvinyltransferase	-1.30	1.76E-17
Cbei_0246	*glmS*	ABR32436.1	Glucosamine--fructose-6-phosphate aminotransferase	-1.25	3.87E-88
Cbei_2591	-	ABR34747.1	Glycosyl transferase family protein	-1.25	3.93E-64
Cbei_2923	-	ABR35066.1	Secretion protein HlyD family protein	-1.20	0.000186472
**Lipid metabolism**					
Cbei_3932	-	ABR36042.1	Alpha/beta fold family hydrolase	-2.44	1.45E-146
[$%]Cbei_0869	-	ABR33053.1	Short-chain dehydrogenase/reductase SDR	-2.14	1.31E-146
Cbei_3630	-	ABR35750.1	Acetyl-CoA acetyltransferase	-2.11	6.32E-60
[Σ]Cbei_3278	-	ABR35407.1	Coenzyme A transferase	-1.90	1.09E-106
∂Cbei_0297	*ispF*	ABR32485.1	2-C-methyl-D-erythritol 2,4-cyclo diphosphate synthase	-1.71	1.26E-58
Cbei_2654	*atoD*	ABR34807.1	3-oxoacid CoA-transferase subunit A	-1.65	3.55E-36
Cbei_3997	-	ABR36107.1	Alpha/beta fold family hydrolase	-1.43	1.82E-26
Cbei_3180	-	ABR35310.1	Short-chain dehydrogenase/reductase SDR	-1.30	2.29E-06
Cbei_1961	-	ABR34131.1	Short-chain dehydrogenase/reductase SDR	-1.20	1.42E-08
Cbei_2653	*ctfB1*	ABR34806.1	3-oxoacid CoA-transferase subunit B	-1.20	6.92E-40
**Cell cycle control and mitosis**					
Cbei_3268	*hol*	ABR35397.1	Holin/phage phi LC3	-4.90	3.35E-145
Cbei_2070	*cotJB*	ABR34238.1	Spore coat peptide assembly protein CotJB	-3.20	2.23E-218
Cbei_0383	-	ABR32571.1	Spore coat protein CotS	-2.53	3.33E-47
Cbei_0385	-	ABR32573.1	Spore coat protein CotS	-2.20	4.23E-49
Cbei_3080	-	ABR35217.1	Small acid-soluble spore protein, alpha/beta type	-1.81	5.89E-102

Cbei_4610	*tlp*	ABR36718.1	Small acid-soluble spore protein Tlp	-1.75	6.61E-12
Cbei_2471	-	ABR34629.1	Small acid-soluble spore protein, alpha/beta type	-1.61	7.63E-109
Cbei_3111	-	ABR35248.1	Small acid-soluble spore protein, alpha/beta type	-1.56	2.87E-07
Cbei_1274	*ytaF*	ABR33454.1	Sporulation protein YtaF	-1.48	1.21E-72
Cbei_3275	-	ABR35404.1	Small acid-soluble spore protein, alpha/beta type	-1.45	1.06E-87
Cbei_1650	-	ABR33824.1	TP901 family phage tail tape measure protein	-1.37	1.96E-46
Cbei_3388	-	ABR35514.1	Tail fiber-like protein	-1.23	3.63E-77
Cbei_3397	-	ABR35523.1	Phage-like element pbsx protein XkdM	-1.21	2.09E-91
Cbei_2345	-	ABR34505.1	Small acid-soluble spore protein, alpha/beta type	-1.20	1.01E-60
**Stress response**					
Cbei_3744	-	ABR35861.1	UviB-like protein	-3.00	7.45E-27
Cbei_2109	*arsD*	ABR34276.1	Arsenical resistance operon trans-acting repressor ArsD	-2.54	6.39E-18
Cbei_3329	-	ABR35456.1	Thioredoxin domain-containing protein	-2.22	5.48E-65
Cbei_2222	-	ABR34387.1	Multi antimicrobial extrusion protein MatE (MATE efflux family protein)	-1.77	3.88E-13
Cbei_2110	*arsA*	ABR34277.1	Arsenite-activated ATPase ArsA	-1.62	6.90E-95
Cbei_1622	-	ABR33796.1	Beta-lactamase domain protein	-1.56	1.86E-10
Cbei_3445	-	ABR35570.1	Small multidrug resistance protein	-1.30	2.72E-06
Cbei_0023	*yqfA*	ABR32213.1	Hemolysin III family channel protein	-1.27	2.42E-58
**Unknown functions**					
Cbei_3718	-	ABR35835.1	Conserved hypothetical protein	-5.72	0.0000
Cbei_1888	-	ABR34059.1	Hypothetical protein	-4.70	1.32E-80
Cbei_3021	-	ABR35160.1	Hypothetical protein	-4.44	0.00
Cbei_2583	-	ABR34739.1	Hypothetical protein	-4.20	0.00
Cbei_1909	-	ABR34079.1	Hypothetical protein/predicted membrane protein	-4.20	0.0000
Cbei_3283	-	ABR35411.1	Hypothetical protein	-4.20	0.0000
Cbei_3285	-	ABR35413.1	Conserved hypothetical protein	-4.13	0.0000
Cbei_3412	-	ABR35538.1	Conserved hypothetical protein	-4.10	0.0000
Cbei_2584	-	ABR34740.1	Hypothetical protein	-4.03	2.14E-134
Cbei_1930	-	ABR34100.1	Hypothetical protein	-4.03	0.0000
Cbei_4714	-	ABR36822.1	Hypothetical protein	-4.02	0.0000
Cbei_1915	-	ABR34085.1	Hypothetical protein	-4.00	4.59E-83
Cbei_1332	-	ABR33512.1	Conserved hypothetical protein	-3.91	1.39E-11
Cbei_0948	-	ABR33132.1	Conserved hypothetical protein	-3.90	0.0000
Cbei_1882	-	ABR34053.1	Hypothetical protein	-3.82	0.0000
Cbei_1863	-	ABR34034.1	Hypothetical protein	-3.82	0.0000
Cbei_1333	-	ABR33513.1	Conserved hypothetical protein	-3.72	1.12E-103
Cbei_5052	-	ABR37158.1	Conserved hypothetical protein	-3.71	0.0000
Cbei_0979	-	ABR33163.1	Conserved hypothetical protein	-3.53	0.0000
Cbei_1680	-	ABR33853.1	Hypothetical protein	-3.50	0.0000
Cbei_3195	-	ABR35325.1	Conserved hypothetical protein	-3.50	8.77E-136
Cbei_3385	-	ABR35511.1	Conserved hypothetical protein	-3.30	7.76E-39
Cbei_3194	-	ABR35324.1	Hypothetical protein	-3.12	3.81E-57
Cbei_3061	-	ABR35199.1	Conserved hypothetical protein	-3.10	0.0000
Cbei_2939	-	ABR35081.1	Hypothetical protein	-3.10	9.64E-74

Cbei_1404	-	ABR33582.1	Conserved hypothetical protein	-3.10	0.0000
Cbei_2506	-	ABR34662.1	Hypothetical protein	-3.10	1.39E-260
Cbei_3334	-	ABR35461.1	Hypothetical protein	-3.10	9.88E-88
Cbei_2071	-	ABR34239.1	Hypothetical protein	-3.10	0.0000
Cbei_2981	-	ABR35121.1	Hypothetical protein	-3.00	5.38E-09
Cbei_4715	-	ABR36823.1	Hypothetical protein	-3.00	2.07E-221
Cbei_2998	-	ABR35137.1	Hypothetical protein	-3.00	1.46E-29
Cbei_2605	-	ABR34761.1	Hypothetical protein	-3.00	0.0000
Cbei_1469	-	ABR33647.1	Conserved hypothetical protein	-2.93	4.56E-48
Cbei_3933	-	ABR36043.1	Hypothetical protein	-2.92	2.16E-43
Cbei_2952	-	ABR35093.1	Hypothetical protein	-2.90	2.35E-06
Cbei_2420	-	ABR34578.1	Putative cell wall binding repeat-containing protein	-2.84	7.60E-97
Cbei_1334	-	ABR33514.1	Hypothetical protein	-2.82	1.74E-218
Cbei_3730	-	ABR35847.1	Hypothetical protein	-2.81	1.78E-100
Cbei_3201	-	ABR35331.1	Phage replisome organizer, putative	-2.80	1.62E-13
Cbei_4700	-	ABR36808.1	Hypothetical protein	-2.80	3.30E-208
Cbei_2606	-	ABR34762.1	Hypothetical protein	-2.80	4.73E-263
Cbei_2938	-	ABR35080.1	Hypothetical protein	-2.80	8.24E-35
Cbei_3302	-	ABR35429.1	Putative post-translational modification; protein turnover; chaperone functions	-2.80	0.0000
Cbei_3457	-	ABR35582.1	Hypothetical protein	-2.71	0.000810884
Cbei_1374	-	ABR33554.1	Hypothetical protein	-2.70	0.04683353
Cbei_0333	-	ABR32521.1	Hypothetical protein	-2.64	0.015607174
Cbei_4999	-	ABR37105.1	Hypothetical protein	-2.61	0.000112945
Cbei_3200	-	ABR35330.1	Conserved hypothetical protein	-2.60	2.33E-22
Cbei_1880	-	ABR34051.1	Conserved hypothetical protein	-2.52	3.95E-208
Cbei_1438	-	ABR33616.1	Conserved hypothetical protein	-2.50	2.69E-122
Cbei_3477	-	ABR35601.1	Conserved hypothetical protein	-2.50	2.64E-25
Cbei_0384	-	ABR32572.1	Hypothetical protein	-2.50	2.60E-98
Cbei_3401	-	ABR35527.1	Hypothetical protein	-2.43	9.16E-131
Cbei_1787	-	ABR33958.1	Hypothetical protein	-2.40	6.13E-173
Cbei_1357	-	ABR33537.1	Hypothetical protein	-2.40	0.006809756
Cbei_4471	-	ABR36580.1	Hypothetical protein	-2.40	1.52E-08
Cbei_1460	-	ABR33638.1	Hypothetical protein	-2.33	1.05E-28
Cbei_2607	-	ABR34763.1	Hypothetical protein	-2.33	2.34E-35
Cbei_1997	-	ABR34167.1	Hypothetical protein	-2.32	0.004069352
Cbei_3733	-	ABR35850.1	Hypothetical protein	-2.31	3.67E-99
Cbei_4922	-	ABR37028.1	Hypothetical protein	-2.30	5.97E-157
Cbei_3384	-	ABR35510.1	Hypothetical protein	-2.30	2.02E-36
Cbei_1908	-	ABR34078.1	Hypothetical protein	-2.30	3.59E-127
Cbei_3946	-	ABR36056.1	Hypothetical protein	-2.20	3.56E-16
Cbei_3149	-	ABR35282.1	Hypothetical protein	-2.20	3.80E-06
Cbei_2433	-	ABR34591.1	Hypothetical protein	-2.20	6.95E-25
Cbei_5000	-	ABR37106.1	Hypothetical protein	-2.20	1.18E-06
Cbei_3399	-	ABR35525.1	Hypothetical protein	-2.13	1.94E-145
Cbei_1649	-	ABR33823.1	Phage XkdN-like protein	-2.13	7.43E-11
Cbei_2570	-	ABR34726.1	Hypothetical protein	-2.11	7.75E-139
Cbei_4355	-	ABR36465.1	Conserved hypothetical protein	-2.10	8.87E-12
Cbei_3723	-	ABR35840.1	Hypothetical protein	-2.10	5.21E-38
Cbei_3731	-	ABR35848.1	Hypothetical protein	-2.06	1.26E-100
Cbei_1508	-	ABR33684.1	Hypothetical protein	-2.05	2.70E-173
Cbei_2308	-	ABR34468.1	Conserved hypothetical protein	-2.04	3.36E-16

Cbei_2571	-	ABR34727.1	Hypothetical protein	-2.04	5.45E-117
Cbei_3066	-	ABR35204.1	Conserved hypothetical protein	-2.02	5.60E-41
Cbei_5030	-	ABR37136.1	Hypothetical protein	-2.00	1.14E-26
Cbei_2349	-	ABR34509.1	Hypothetical protein	-2.00	1.01E-179
Cbei_1644	-	ABR33818.1	Hypothetical protein	-1.94	2.25E-20
Cbei_2291	-	ABR34451.1	Hypothetical protein	-1.93	4.74E-23
Cbei_1619	-	ABR33793.1	Conserved hypothetical protein	-1.93	5.04E-25
Cbei_1827	-	ABR33998.1	Hypothetical protein	-1.93	1.30E-301
Cbei_1664	-	ABR33838.1	Conserved hypothetical protein	-1.93	0.007014092
Cbei_3286	-	ABR35414.1	Hypothetical protein	-1.90	9.93E-18
Cbei_1646	-	ABR33820.1	Hypothetical protein	-1.90	0.005917711
Cbei_4987	-	ABR37093.1	Hypothetical protein	-1.90	5.07E-46
Cbei_3734	-	ABR35851.1	Hypothetical protein	-1.90	3.15E-43
Cbei_2600	-	ABR34756.1	Conserved hypothetical protein	-1.83	1.04E-181
Cbei_1654	-	ABR33828.1	Hypothetical protein	-1.83	2.27E-14
Cbei_1655	-	ABR33829.1	Hypothetical protein	-1.82	3.06E-15
Cbei_1656	-	ABR33830.1	Baseplate J family protein	-1.81	4.02E-33
Cbei_3738	-	ABR35855.1	YibE/F family protein	-1.81	6.22E-27
Cbei_3305	-	ABR35432.1	Conserved hypothetical protein	-1.81	2.68E-07
Cbei_1651	-	ABR33825.1	Hypothetical protein	-1.80	1.96E-102
Cbei_1658	-	ABR33832.1	phage-like element pbsx protein XkdT	-1.80	1.18E-13
Cbei_0895	-	ABR33079.1	Hypothetical protein	-1.80	0.000137308
Cbei_3994	-	ABR36104.1	Conserved hypothetical protein	-1.77	2.41E-20
Cbei_3398	-	ABR35524.1	phage-like element pbsx protein XkdK	-1.75	4.36E-232
Cbei_1255	*yjlA*	ABR33436.1	Hypothetical protein	-1.75	1.01E-36
Cbei_1810	-	ABR33981.1	Hypothetical protein	-1.74	8.48E-23
Cbei_3705	-	ABR35822.1	Hypothetical protein	-1.71	6.67E-31
Cbei_3478	-	ABR35602.1	Hypothetical protein	-1.71	1.66E-33
Cbei_1640	-	ABR33814.1	Hypothetical protein	-1.70	0.010321003
Cbei_3396	-	ABR35522.1	Phage XkdN-like protein	-1.70	5.26E-190
Cbei_1636	-	ABR33810.1	Conserved hypothetical protein	-1.67	2.29E-47
Cbei_3745	-	ABR35862.1	Hypothetical protein	-1.66	0.000163246
Cbei_3263	-	ABR35392.1	Hypothetical protein	-1.66	2.82E-101
Cbei_1998	-	ABR34168.1	Hypothetical protein	-1.66	1.32E-11
Cbei_1905	-	ABR34076.1	Hypothetical protein	-1.64	3.16E-07
Cbei_1647	-	ABR33821.1	Hypothetical protein	-1.60	9.59E-32
Cbei_3387	-	ABR35513.1	Conserved hypothetical protein	-1.56	1.47E-86
Cbei_1657	-	ABR33831.1	Hypothetical protein (amino acid transport)	-1.53	1.23E-56
Cbei_3256	-	ABR35385.1	Hypothetical protein	-1.56	0.002448192
Cbei_2679	-	ABR34832.1	Conserved hypothetical protein	-1.54	1.01E-84
Cbei_3550	-	ABR35672.1	Conserved hypothetical protein	-1.53	2.73E-11
Cbei_1660	-	ABR33834.1	Hypothetical protein	-1.52	0.000124408
Cbei_1567	-	ABR33741.1	Conserved hypothetical protein	-1.50	1.36E-30
Cbei_1645	-	ABR33819.1	Hypothetical protein	-1.50	9.69E-34
Cbei_2791	-	ABR34941.1	Hypothetical protein	-1.50	0.000292501
Cbei_1916	-	ABR34086.1	Hypothetical protein	-1.46	6.37E-27
Cbei_1296	-	ABR33476.1	Hypothetical protein	-1.45	2.49E-05
Cbei_2636	-	ABR34790.1	Hypothetical protein	-1.45	2.68E-09
Cbei_2552	-	ABR34708.1	Hypothetical protein	-1.44	6.60E-55
Cbei_3725		ABR35842.1	Domain of unknown function DUF1906	-1.44	5.49E-66

Cbei_1653	-	ABR33827.1	Hypothetical protein	-1.44	1.15E-17
Cbei_1637	-	ABR33811.1	Hypothetical protein	-1.42	1.04E-37
Cbei_1638	-	ABR33812.1	Hypothetical protein	-1.42	1.55E-29
Cbei_1643	-	ABR33817.1	Hypothetical protein	-1.41	8.28E-25
Cbei_1624	-	ABR33798.1	Hypothetical protein	-1.41	5.48E-08
Cbei_3225	-	ABR35355.1	Hypothetical protein	-1.40	0.003996084
Cbei_1639	-	ABR33813.1	Hypothetical protein	-1.40	3.26E-28
Cbei_1642	-	ABR33816.1	Hypothetical protein	-1.40	1.14E-09
Cbei_3294	-	ABR35421.1	Hypothetical protein	-1.40	4.32E-06
Cbei_3247	-	ABR35376.1	Hypothetical protein	-1.37	7.18E-34
Cbei_1617	-	ABR33791.1	Hypothetical protein	-1.36	0.003409005
Cbei_1870	-	ABR34041.1	Hypothetical protein	-1.35	1.07E-10
Cbei_3921	-	ABR36033.1	Hypothetical protein	-1.35	6.54E-10
Cbei_1928	-	ABR34098.1	Hypothetical protein	-1.34	8.19E-12
Cbei_0896	-	ABR33080.1	Hypothetical protein	-1.33	4.38E-17
Cbei_3391	-	ABR35517.1	Hypothetical protein	-1.31	4.56E-52
Cbei_3381	-	ABR35507.1	Conserved hypothetical protein	-1.31	0.010749049
Cbei_2223	-	ABR34388.1	Conserved hypothetical protein	-1.31	1.85E-07
Cbei_1628	-	ABR33802.1	Hypothetical protein	-1.31	0.007508957
Cbei_2409	-	ABR34567.1	Hypothetical protein	-1.31	6.12E-06
Cbei_3319	-	ABR35446.1	Hypothetical protein	-1.30	1.76E-30
Cbei_3742	-	ABR35859.1	Hypothetical protein	-1.30	6.33E-07
Cbei_3408	-	ABR35534.1	Hypothetical protein	-1.27	0.003298174
Cbei_3729	-	ABR35846.1	Hypothetical protein	-1.27	2.88E-34
Cbei_1503	-	ABR33679.1	Hypothetical protein	-1.26	2.37E-05
Cbei_1042	-	ABR33226.1	Hypothetical protein	-1.26	5.39E-17
Cbei_1865	-	ABR34036.1	Hypothetical protein	-1.25	5.54E-13
Cbei_4372	-	ABR36482.1	Hypothetical protein	-1.24	0.000429523
Cbei_4697	-	ABR36805.1	Zn-finger containing protein	-1.24	4.36E-07
Cbei_1779	-	ABR33950.1	Conserved hypothetical protein	-1.24	1.68E-33
Cbei_3297	-	ABR35424.1	Hypothetical protein	-1.22	0.023093382
Cbei_4770	-	ABR36877.1	Hypothetical protein	-1.22	3.15E-48
Cbei_3392	-	ABR35518.1	Hypothetical protein	-1.20	1.12E-57
Cbei_2640	-	ABR34794.1	Hypothetical protein	-1.20	3.68E-07
Cbei_3205	-	ABR35335.1	Hypothetical protein	-1.20	7.43E-14
Cbei_0897	-	ABR33081.1	Hypothetical protein	-1.20	8.88E-16
Cbei_2633	-	ABR34787.1	Hypothetical protein	-1.20	1.54E-06
Cbei_3713	-	ABR35830.1	Hypothetical protein	-1.20	1.97E-25
Cbei_0646	-	ABR32833.1	Hypothetical protein	-1.20	3.33E-07
